# Glucosylceramide is essential for Heartland and Dabie bandavirus glycoprotein-induced membrane fusion

**DOI:** 10.1371/journal.ppat.1011232

**Published:** 2023-03-15

**Authors:** Tian Xia, Xin Wu, Eunjin Hong, Kyle Jung, Chih-Jen Lai, Mi-Jeong Kwak, Hogyu Seo, Stephanie Kim, Zhongyi Jiang, Inho Cha, Jae U. Jung

**Affiliations:** 1 Department of Cancer Biology, Infection Biology Program, and Global Center for Pathogen and Human Health Research, Lerner Research Institute, Cleveland Clinic, Cleveland, Ohio, United States of America; 2 Department of Molecular Microbiology and Immunology, Keck School of Medicine, University of Southern California, California, United States of America; Icahn School of Medicine at Mount Sinai, UNITED STATES

## Abstract

Due to climate changes, there has been a large expansion of emerging tick-borne zoonotic viruses, including *Heartland bandavirus* (HRTV) and *Dabie bandavirus* (DBV). As etiologic agents of hemorrhagic fever with high fatality, HRTV and DBV have been recognized as dangerous viral pathogens that likely cause future wide epidemics. Despite serious health concerns, the mechanisms underlying viral infection are largely unknown. HRTV and DBV Gn and Gc are viral surface glycoproteins required for early entry events during infection. Glycosphingolipids, including galactosylceramide (GalCer), glucosylceramide (GlcCer) and lactosylceramide (LacCer), are a class of membrane lipids that play essential roles in membrane structure and viral lifecycle. Here, our genome-wide CRISPR/Cas9 knockout screen identifies that glycosphingolipid biosynthesis pathway is essential for HRTV and DBV infection. The deficiency of UDP-glucose ceramide glucosyltransferase (UGCG) that produces GlcCer resulted in the loss of infectivity of recombinant viruses pseudotyped with HRTV or DBV Gn/Gc glycoproteins. Conversely, exogenous supplement of GlcCer, but not GalCer or LacCer, recovered viral entry of UGCG-deficient cells in a dose-dependent manner. Biophysical analyses showed that GlcCer targeted the lipid-head-group binding pocket of Gc to form a stable protein-lipid complex, which allowed the insertion of Gc protein into host lysosomal membrane lipid bilayers for viral fusion. Mutagenesis showed that D841 residue at the Gc lipid binding pocket was critical for GlcCer interaction and thereby, viral entry. These findings reveal detailed mechanism of GlcCer glycosphingolipid in HRTV and DBV Gc-mediated membrane fusion and provide a potential therapeutic target for tickborne virus infection.

## Author summary

*Heartland bandavirus* (HRTV) and *Dabie bandavirus* (DBV) were recently identified as emerging tick-borne zoonotic viruses in the United States and Asia, respectively. As etiologic agents of hemorrhagic fever with high fatality, HRTV and DBV have been recognized as dangerous viral pathogens that likely cause future wide epidemics. Despite serious health concerns, the mechanisms underlying viral infection are largely unknown. Here, we use genome-wide CRISPR/Cas9 knockout screens to determine the requirements of HRTV entry in mammalian cells. We found that the glycosphingolipid biosynthesis pathway is essential for HRTV and DBV infection. The infectivity of HRTV and DBV in glycosphingolipid biosynthesis-deficient cells was drastically reduced. We also found that glucosylceramide (GlcCer) plays a vital role in HRTV glycoproteins-mediated membrane fusion. The GlcCer targets the lipid-head-group binding pocket of HRTV glycoprotein in the host lysosomal membrane to form a stable lipid-protein complex, thereby facilitating viral fusion and entry. Our study reveals the detailed molecular mechanism of GlcCer glycosphingolipid in HRTV and DBV Gc-mediated membrane fusion and provides a potential therapeutic target for tick-borne virus infection.

## Introduction

Emerging pathogenic tick-borne viruses (TBVs) have recently attracted wide attention due to their considerable impact on public health [1]. *Heartland bandavirus* (HRTV), a member of the *Bandavirus* genus of the *Phenuiviridae* family, has been isolated from the lone-star tick (*Amblyomma americanum*) in the United States [2]. *Phenuiviridae* family contains several highly pathogenic viruses, such as *Dabie bandavirus* (DBV; also called Severe Fever with Thrombocytopenia Syndrome virus, SFTSV) and Rift Valley fever virus (RVFV)[3]. HRTV is genetically closely related to DBV isolated from Asian longhorned tick (*Haemaphysalis longicornis)* in Eastern Asia [4]. Particularly, the recent spread of this Asian longhorned tick to over 20 states of USA increase the potential for outbreaks of viral disease beyond the Far East Asia [5]. Patients infected with HRTV and DBV have many similar clinical manifestations, including hemorrhagic fever, thrombocytopenia, leukopenia, multi-organ failure, and death [6, 7]. Currently, no drugs or vaccines are approved for therapy or prevention of HRTV and DBV infection. Therefore, detailed study is in desperate need to understand the mechanisms of viral entry and replication.

The *Bandavirus* is an enveloped virus genus that possesses a tri-segmented genome composed of either negative-sense or ambi-sense RNA [8]. The large (L) segment encodes the RNA-dependent RNA polymerase (RdRp), which is essential for viral transcription and replication [9]. The small (S) segment encodes the nucleocapsid (N) protein and the non-structural (NS) protein which can form the ribonucleoprotein complexes (RNPs) with its RNA genome and regulate host immune response, respectively [10, 11]. The medium (M) segment encodes a glycoprotein precursor Gn/Gc, which is processed into Gn and Gc proteins by host protease [12]. The amino-acid sequence of HRTV glycoproteins shows 62% and 24% identity to those of DBV and RVFV, respectively. Based on the structure of RVFV virion, the Gn and Gc are organized as heterodimers and form highly ordered capsomers on the viral surface [13]. During viral entry, Gn and Gc proteins are responsible for receptor binding and membrane fusion [14]. However, the precise mechanisms governing the entry of HRTV and DBV into host cells require additional investigation.

The entry of HRTV and DBV is initiated by the interaction between glycoproteins and cell-surface receptors or attachment factors. Several host factors have been identified as entry factors for *Phenuiviridae* family viruses, including DC-SIGN, L-SIGN, NMMHC-IIA and Lrp1[15–18]. After binding to receptors, viral particles are internalized by endocytic machinery. Subsequently, virions are sorted into endosomal vesicles, transported from early endosome to late endosome and then to lysosome [19]. In the late endosome and lysosome, Gc protein undergoes a low pH-triggered conformational change, thereby exposing its hydrophobic peptides called the fusion loops. As a class II fusion protein, Gc then inserts into the endosomal membrane *via* its fusion loops and induces membrane fusion [20]. Ultimately, viral RNPs are released into the cytoplasm for viral replication.

Sphingolipids are a class of membrane lipids that include ceramide, sphingomyelin, and hundreds of different glycosphingolipids (GSLs)[21]. In eukaryotic cells, sphingolipids comprise 10–20% of plasma membrane lipids and play essential roles in cellular membrane structure and signaling [22]. In addition, recent studies indicate that sphingolipids are involved in multiple steps of viral life cycle, including attachment and entry, intracellular transport, replication, viral assembly, and budding [23, 24]. For instance, galactosylceramide (GalCer) interacts with human immunodeficiency virus (HIV) envelope protein gp120 in the plasma membrane to facilitate its entry into CD4^-^ cells [25, 26]; glucosylceramide (GlcCer) is required to regulate influenza A virus (IAV) trafficking along the endocytic pathway, which is essential for viral fusion [27]; gangliosides are required for hepatitis A virus (HAV) entry as endosomal receptors [28]. Recently, a study has shown that UDP-glucose ceramide glucosyltransferase (UGCG) is essential for efficient DBV entry [29]. However, the mechanistic role of sphingolipids in bandavirus infection is still unclear.

Genome-wide CRISPR/Cas9 screens have enabled the identification of host factors required for efficient virus infection. Here, we performed a cell survival-based screening with HRTV infection in 293T cells transduced with a single-guide RNAs (sgRNAs) lentiviral library. This showed that deficiency in the sphingolipid biosynthesis pathway significantly inhibited HRTV entry. HRTV Gc protein required GlcCer for low pH-induced membrane fusion in the lysosome. In addition, the lipid-head-group binding pocket of Gc interacted with GlcCer to form a stable protein-membrane complex. This study reveals detailed mechanism of GlcCer-mediated entry for HRTV and DBV.

## Result

### Glucosylceramide biosynthesis pathway is essential for HRTV infection

**Fig 1:**
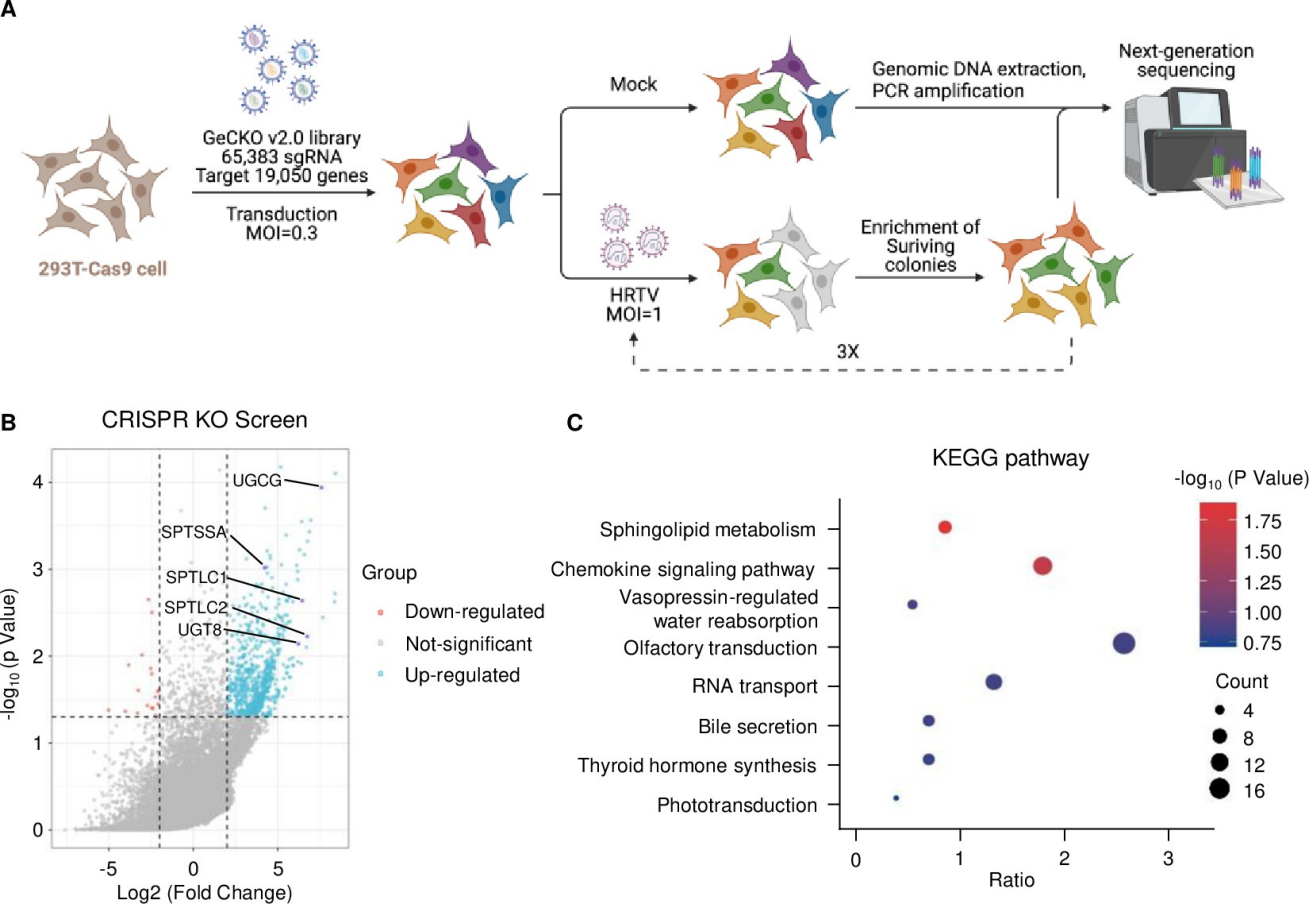
Genome-wide CRISPR screen identifies HRTV entry factors. **A.** Schematic of genome-wide CRISPR/Cas9 KO screen in 293T cells. Figure created with BioRender.com. **B.** Volcano plot analysis of the CRISPR/Cas9 KO screen results. Each dot represents a unique gene identified from sequencing results. The labeled genes are involved in the glycosphingolipid biosynthesis pathway. **C.** Bubble plot showing the pathway analysis of the statistically enriched genes in the screen. Bubble size and color reflect the statistical significance and the number of genes enriched in the relevant pathway.

To identify host factors required for HRTV infection, we performed a genome-wide CRISPR/Cas9 knockout screen in 293T-Cas9 cells (Fig 1A). We transduced 293T-Cas9 cells with genome-scale CRISPR knockout (GeCKO) lentiviral pooled libraries that contain 65,383 sgRNA and target 19,050 human genes [30]. After transduction, library cells were selected with puromycin and pooled together. Approximately 6x10^7^ gene-edited cells were infected with HRTV at a multiplicity of infection (MOI) of 1, and virus infection-induced cytopathic effect was visible five days after infection. Surviving cells were reseeded for two additional rounds of HRTV infection. Genomic DNAs were extracted from uninfected control cells and surviving cells from HRTV infection, and the integrated sgRNA sequences were amplified by PCR and subjected to next-generation sequencing. The enrichment of each sgRNA was analyzed by MAGeCK, and the results were plotted on a volcano map (Fig 1B and S1 File). A total of 755 genes were statistically enriched (p<0.05 and fold change>4). The Kyoto Encyclopedia of Genes and Genomes (KEGG) pathway analysis revealed that the sphingolipid metabolism pathway was highlighted in HRTV infection screen (Fig 1C).

**Fig 2:**
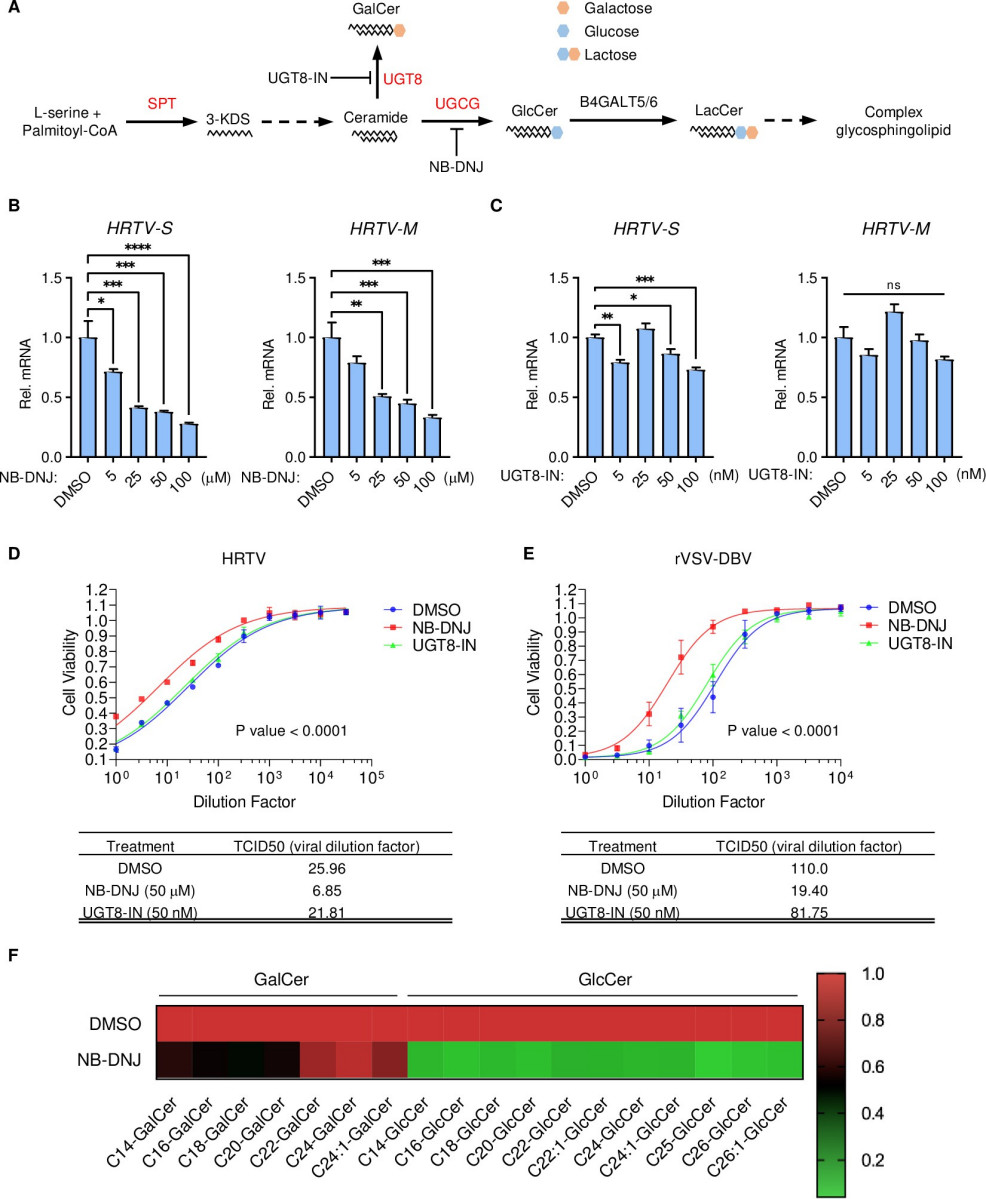
Glucosylceramide biosynthesis pathway is essential for HRTV infection. **A.** Diagram of glycosphingolipid biosynthesis pathway. Genes highlighted in red were identified from CRISPR/Cas9 screen. **B-C.** Effect of NB-DNJ or UGT8-IN on HRTV replication. 293T cells were pre-treated with NB-DNJ or UGT8-IN for 24 hours before infection with HRTV (MOI = 1). Viral RNAs of the S-segment and M-segment were measured by qPCR and normalized to *GAPDH* mRNA. Data shown are means ± SEM from representative experiments (n = 3 technical replicates). *P* values were determined by ordinary one-way ANOVA with Dunnett’s multiple comparison tests. ****, *P* < 0.0001; ***, *P* < 0.001; **, *P* < 0.01; *, *P* < 0.05. **D-E.** Effect of NB-DNJ and UGT8-IN on HRTV or rVSV-DBV infection. To generate a replication-competent rVSV-DBV, the VSV glycoprotein in its genome was replaced with DBV Gn/Gc. After incubation with indicated drugs for 24 hours, half-log serially diluted HRTV or rVSV-DBV was added to 4 replicate wells of a 96-well plate containing 293T cells for 72 hours. TCID_50_ was calculated by plotting cell viability versus dilution factor using GraphPad Prism and fitted by a dose-response (variable slope) algorithm. **F.** Heat map of the levels of intracellular GalCer and GlcCer in 293T cells with or without NB-DNJ treatment.

Sphingolipid *de novo* biosynthesis occurs in the endoplasmic reticulum (ER) by serine palmitoyltransferase (SPT) complex that comprises three proteins: SPTLC1, SPTLC2, and SPTSSA. This complex catalyzes the condensation of L-serine and palmitoyl-CoA to generate sphingolipid precursor 3-ketodihydrosphingosine (3-KDS) that is enzymatically converted to ceramide, the lipid moiety, for all glycosphingolipids (GSLs) (Fig 2A). Ceramide is then converted to GalCer and GlcCer by uridine diphosphate glycosyltransferase 8 (UGT8) in the ER, and UGCG in the Golgi apparatus, respectively. GlcCer is the crucial component for the synthesis of various complex GSLs, such as lactosylceramide (LacCer) and gangliosides. CRISPR/Cas9 screen found that five genes in the sphingolipid synthesis pathway were enriched in surviving cells from HRTV infection, indicating the critical role of sphingolipid biosynthesis for HRTV infection (Fig 1B).

To validate CRISPR/Cas9 screen results, 293T cells were treated with UGCG inhibitor N-butyl-deoxynojirimycin (NB-DNJ) or UGT8 inhibitor (UGT8-IN) upon HRTV infection, and RNA levels of the S-segment or the M-segment were measured by quantitative real-time PCR (qPCR). The results showed that NB-DNJ, but not UGT8-IN, markedly inhibited HRTV replication in a dose-dependent manner (Fig 2B and 2C). The TCID_50_ assay revealed that cells treated with NB-DNJ were resistant to cell death induced by HRTV infection, as well as replication-competent rVSV-DBV pseudotyped virus infection (Fig 2D and 2E). Finally, supercritical fluid chromatography-tandem mass spectrometry (SFC-MS/MS) analysis showed that GlcCer was substantially depleted upon NB-DNJ treatment (Fig 2F and S2 File). These findings indicate that the SPT-UGCG axis is critical for HRTV entry.

### GlcCer is required for the efficient infection of HRTV and DBV

**Fig 3:**
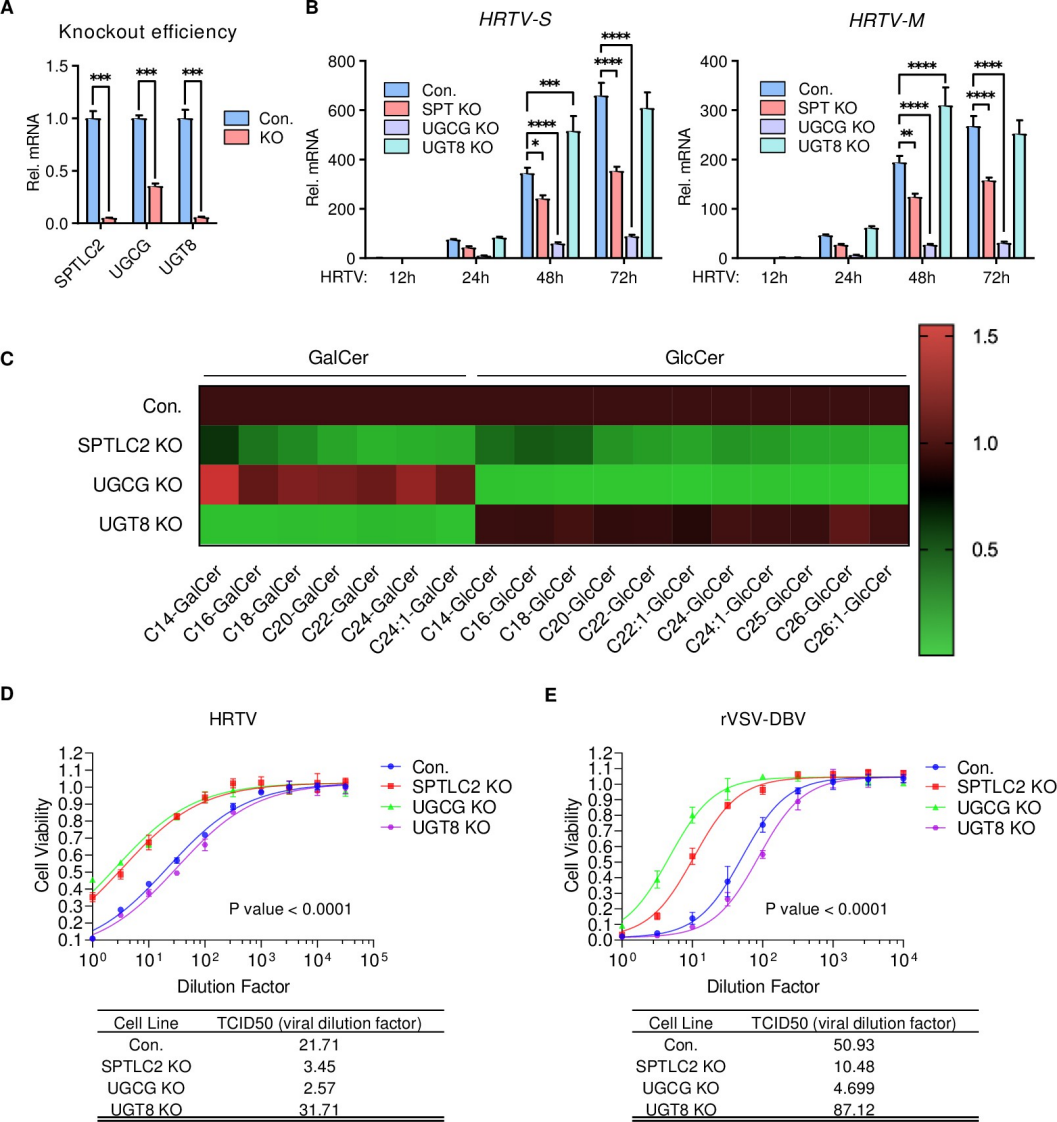
HRTV requires GlcCer for efficient infection. **A-B.** Effect of SPTLC2-, UGCG- or UGT8-deficiency on HRTV replication. Control and KO 293T cells were infected with HRTV (MOI = 1) for the indicated times for qPCR analysis. Knockout efficiency of *SPTLC2-*, *UGCG-* or *UGT8* gene was measured by qPCR. Data shown are means ± SEM from representative experiments (n = 3 technical replicates). *P* values were determined by multiple unpaired *t*-tests (**A**) or two-way ANOVA with Dunnett’s multiple comparison tests (**B**). ****, *P* < 0.0001; ***, *P* < 0.001; **, *P* < 0.01; *, *P* < 0.05. **C.** Heat map of the levels of intracellular GalCer and GlcCer in SPTLC2-, UGCG-, or UGT8-deficient 293T cell lines. **D-E.** TCID_50_ of HRTV and rVSV-DBV in control, SPTLC2-, UGCG-, or UGT8-deficient 293T cells. Half-log serially diluted HRTV or rVSV-DBV was added to 4 replicate wells of a 96-well plate for 72 hours before luminescence measurement.

To further investigate the critical role of GlcCer synthesis pathway in HRTV infection, SPTLC2-deficient (SPT-KO), UGCG-deficient (UGCG-KO), or UGT8-deficient (UGT8-KO) 293T cells were generated by CRISPR/Cas9 knockout approach (Figs 3A and S2–S4). Compared to control cells, UGCG-KO cells showed nearly complete loss of HRTV infection and SPT-KO cells showed detectable inhibition of HRTV infection, but UGT8-KO cells showed little to no inhibition of HRTV infection (Fig 3B). Furthermore, SPT-KO, UGCG-KO, or UGT8-KO HeLa cells also showed the similar results (S1A, S1B and S2–S4 Figs). qPCR analysis demonstrated that HRTV infection was significantly reduced in SPTLC2 or UGCG knockout cells, but not UGT8-KO cells (S1C Fig). Immunofluorescence analysis also showed that the intracellular N protein levels in SPT-KO or UGCG-KO cells were drastically lower than those in control or UGT8-KO cells (S1D and S1E Fig). In contrast, vesicular stomatitis virus (VSV)-GFP infection was not affected in these cells compared to control cells (S1D and S1E Fig). Quantitative SFC-MS/MS analysis showed that the levels of intracellular GlcCer and GalCer in SPT-KO 293T cells were reduced to about 30% of those in control cells and the levels of intracellular GlcCer or GalCer in UGCG-KO or UGT8-KO cells, respectively, were significantly decreased to only 5% of those in control cells (Fig 3C and S2 File). Consistently, TCID_50_ assay showed the resistance of SPT-KO and UGCG-KO cells to HRTV or rVSV-DBV infection (Fig 3D and 3E). These results demonstrate a strong correlation between intracellular GlcCer level and HRTV and DBV infection.

**Fig 4:**
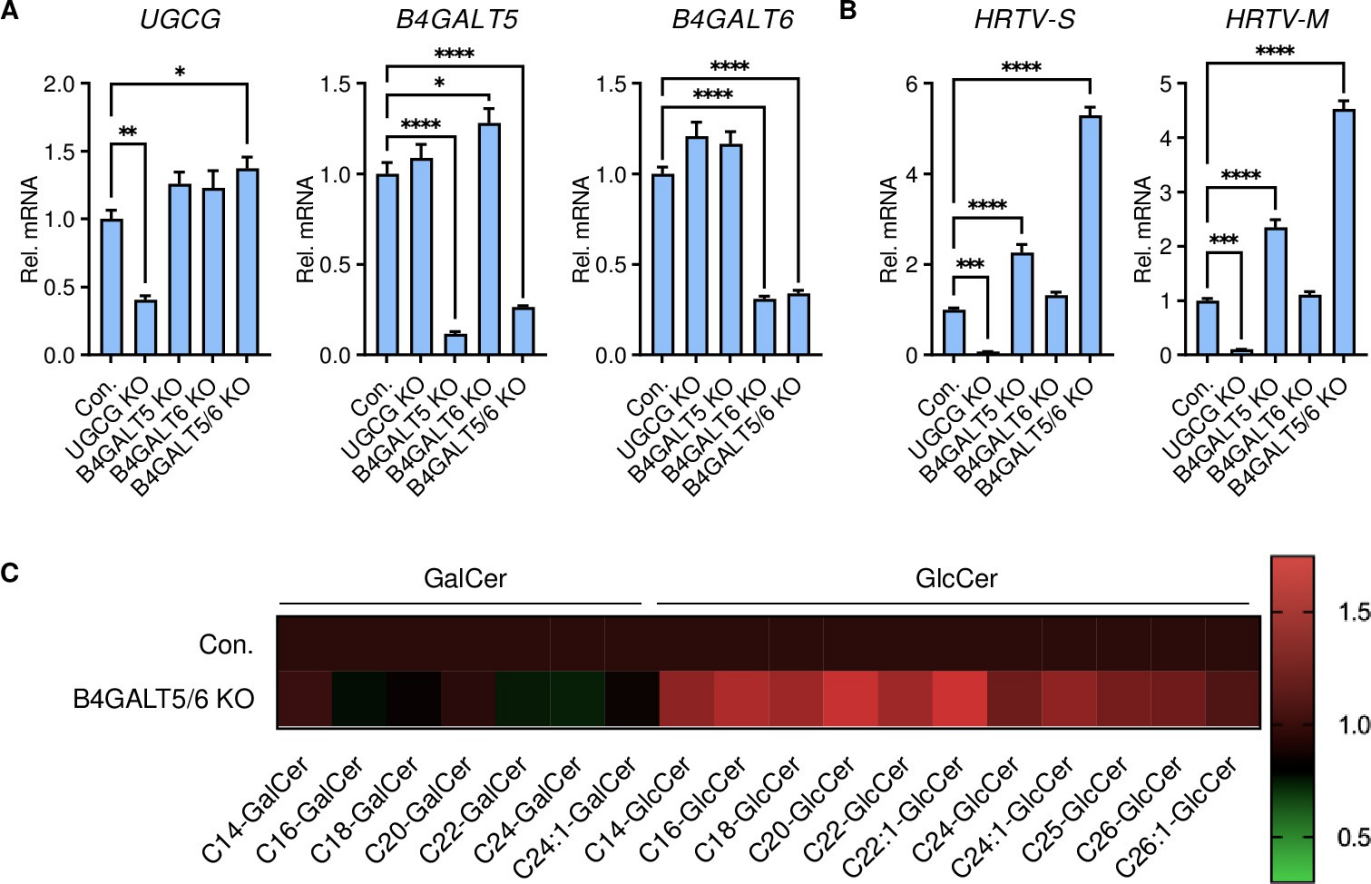
Glycosphingolipid downstream of GlcCer is not required for HRTV infection. **A-B.** Effect of B4GALT5-, B4GALT6- or B4GALT5/6 double-deficiency on HRTV replication. Control and KO 293T cells were infected with HRTV (MOI = 1) for 24 hours, followed by qPCR analysis. Data shown are means ± SEM from representative experiments (n = 3 technical replicates). *P* values were determined by ordinary one-way ANOVA with Dunnett’s multiple comparison tests. ****, *P* < 0.0001; ***, *P* < 0.001; **, *P* < 0.01; *, *P* < 0.05. **C.** Heat map of the levels of intracellular GalCer and GlcCer in control or B4GALT5/6 double knockout 293T cell lines.

LacCer synthase, the downstream enzyme of UGCG, is encoded by *β1*,*4-galactosyltransferase 5* (*B4GALT5*) and *β1*,*4-galactosyltransferase 6* (*B4GALT6*) genes. To determine whether LacCer and other complex GSLs are required for HRTV infection, B4GALT5-deficient (B4GALT5-KO), B4GALT6-deficient (B4GALT6-KO), and B4GALT5/6 double-deficient (B4GALT5/6-DKO) 293T cells were also generated by CRISPR/Cas9 knockout approach (Figs 4A and S5 and S6). Interestingly, compared to those in control cells, HRTV RNA levels were dramatically enhanced in B4GALT5/6-DKO cells and detectably increased in B4GALT5-KO cells (Fig 4B). Furthermore, intracellular GlcCer levels were detectably higher in B4GALT5/6-DKO cells than in control cells (Fig 4C and S2 File). These results indicate that LacCer and its downstream products are not required for HRTV infection, and that the enhanced HRTV infection in B4GALT5/6-DKO cells is likely due to the accumulation of GlcCer.

**Fig 5:**
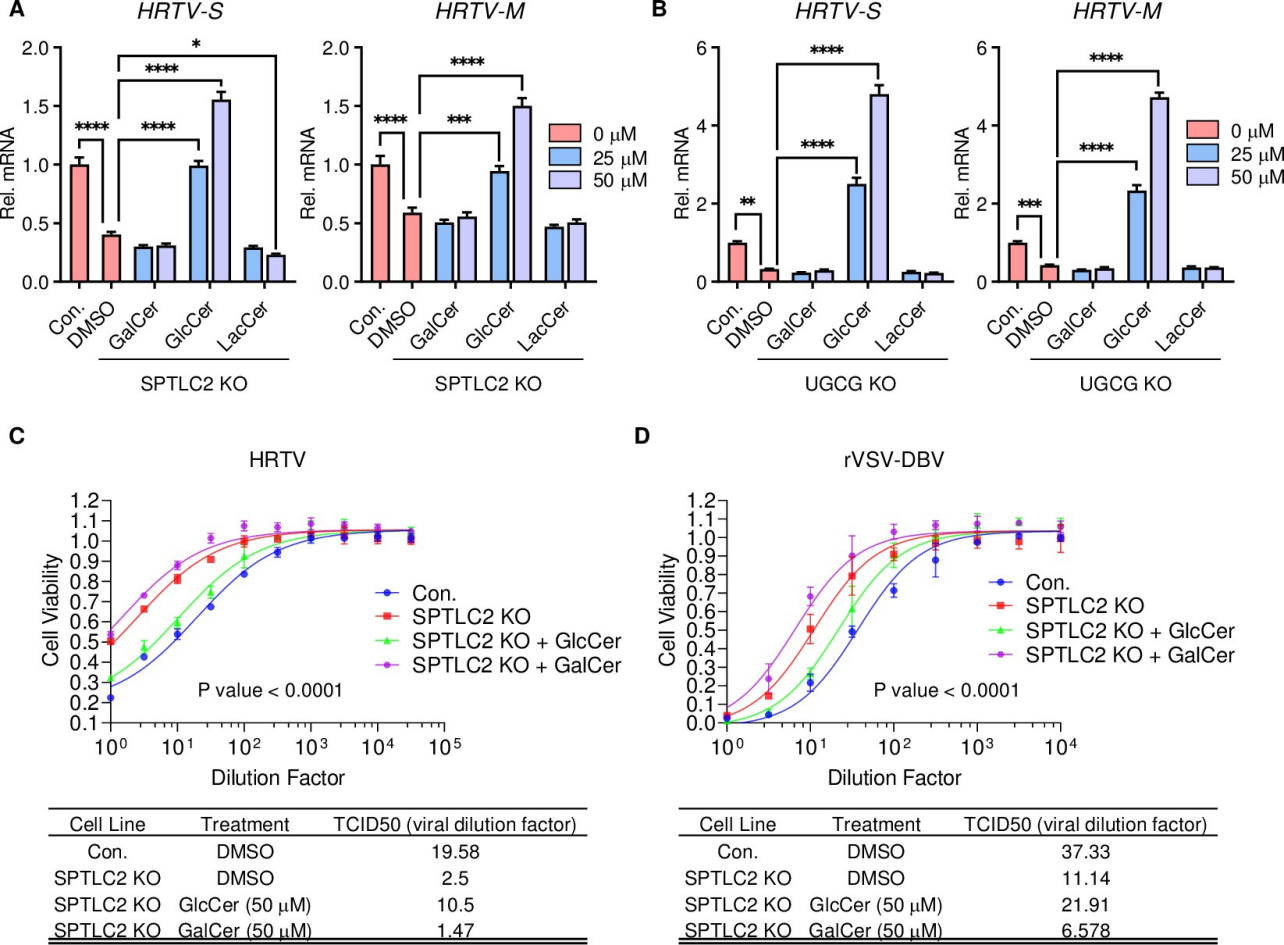
Supplementing with GlcCer enhances HRTV infection. **A-B.** Exogenous glycosphingolipid supplement for HRTV replication in SPTLC2 or UGCG KO 293T cells. Cells were cultured in media supplemented with 0–50 μM indicated glycosphingolipid for 24 hours before HRTV infection (MOI = 1). Viral RNA levels were measured at 24 hours post-infection. Control cells without glycosphingolipid supplement was shown for comparison. Data shown are means ± SEM from representative experiments (n = 3 technical replicates). *P* values were determined by ordinary one-way ANOVA with Dunnett’s multiple comparison tests. ****, *P* < 0.0001; ***, *P* < 0.001; **, *P* < 0.01; *, *P* < 0.05. **C-D.** TCID_50_ of HRTV and rVSV-DBV in SPTLC2 knockout cell with GalCer or GlcCer supplement. Cells were cultured in media supplemented with 50 μM indicated glycosphingolipid for 24 hours before HRTV or rVSV-DBV infection.

To determine whether GlcCer was specific for HRTV infection, GalCer, GlcCer, or LacCer was exogenously supplemented to culture medium of SPT-KO cells or UGCG-KO cells at 24 hours before HRTV infection. This showed that only GlcCer supplement rescued HRTV infection in a dose-dependent manner in both SPT-KO and UGCG-KO cells. However, neither GalCer nor LacCer supplement rescued HRTV infection in those KO cells (Fig 5A and 5B). TCID_50_ assays also showed that GlcCer supplement increased the susceptibility of SPT-KO cells to HRTV or rVSV-DBV infection (Fig 5C and 5D). Collectively, these results show that UGCG and its product GlcCer are essential for HRTV infection.

### GlcCer is critical for HRTV-induced membrane fusion

**Fig 6:**
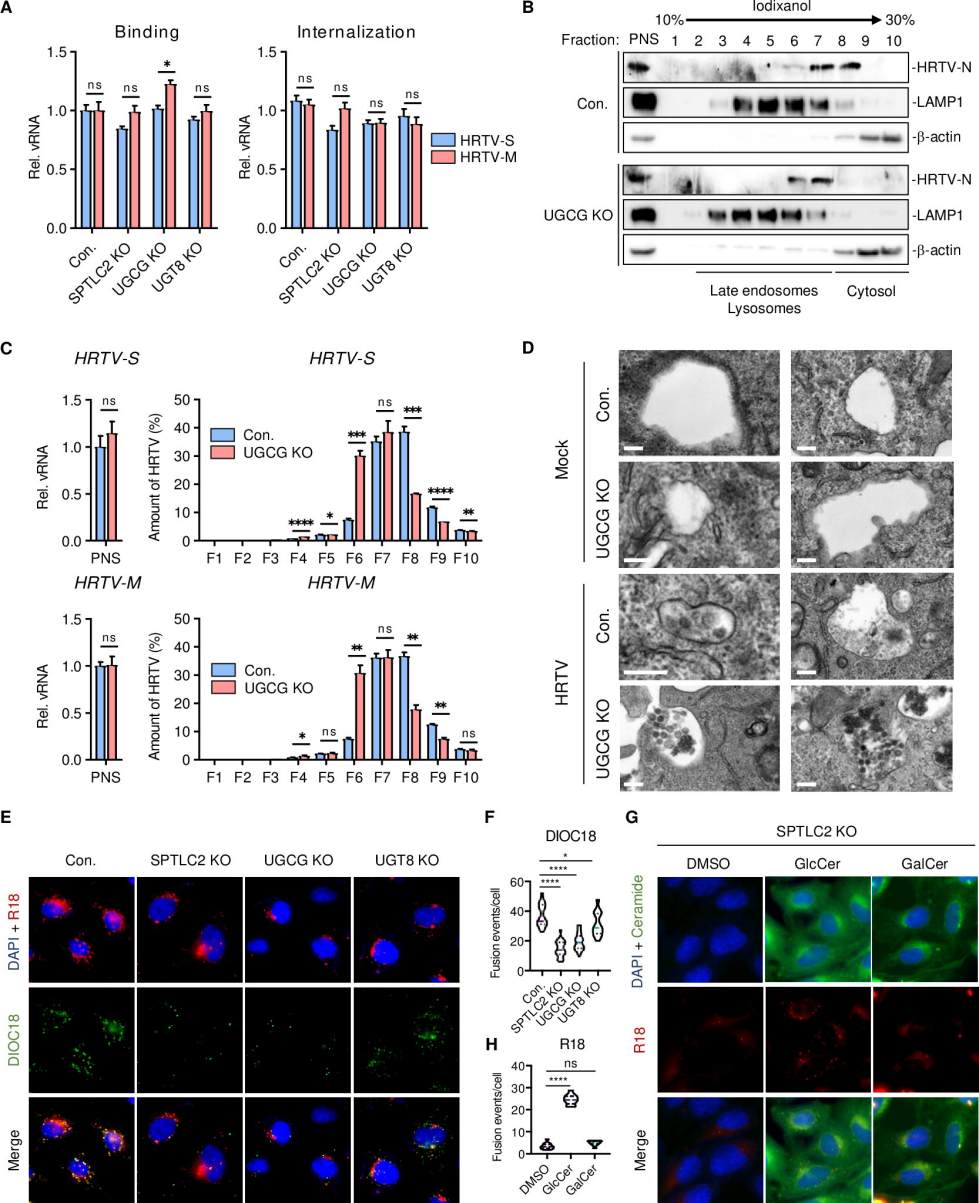
GlcCer is critical for HRTV-induced membrane fusion. **A.** Effect of SPTLC2-, UGCG- or UGT8-deficiency on HRTV binding and internalization. For binding assay, control and KO HeLa cells were incubated with HRTV particles at 4°C for 1 hour. For internalization assay, after 4°C incubation, cells were washed by PBS and then moved to 37°C for additional 2 hours before qPCR assay. Data shown are means ± SEM from representative experiments (n = 3 technical replicates). *P* values were determined by multiple unpaired t-tests. *, *P* < 0.05. **B-C.** Subcellular fractionation of HRTV-infected control or UGCG-KO cells. Cells were treated as described in Materials and Methods, and the PNS was fractionated by Optiprep gradient centrifugation. PNS and each fraction were subjected to immunoblotting analysis with the indicated antibodies (**B**). Total RNAs were extracted from PNS and each fraction for qPCR assay (**C**). Data shown are means ± SEM from representative experiments (n = 3 technical replicates). *P* values were determined by multiple unpaired t-tests. ****, *P* < 0.0001; ***, *P* < 0.001; **, *P* < 0.01; *, *P* < 0.05. **D.** Effect of UGCG-deficiency on HRTV trafficking. Control and UGCG-KO 293T cells were infected with HRTV at MOI = 50 for 4 hours. Cells were processed for thin-section electron microscopy according to standard procedures [47]. Bars, 200nm. **E-F.** Effect of SPTLC2-, UGCG- or UGT8-deficiency on HRTV-induced membrane fusion. HeLa cells were infected with DiOC18/R18-labeled HRTV for 30 min at 4°C and then incubated at 37°C for 2 hours (**E**). The numbers of fluorescent DiOC18 puncta were quantified by fluorescence microscopy (**F**). *P* values were determined by ordinary one-way ANOVA with Dunnett’s multiple comparison test (n  =  15). ****, *P* < 0.0001; *, *P* < 0.05. **G-H.** Effect of exogenous GlcCer and GalCer supplement of SPTLC2-KO cells on HRTV-induced membrane fusion. HeLa cells were cultured in media supplemented with 50 μM NBD-labeled GlcCer or GalCer for 24 hours. Cells were subsequently incubated with R18-labeled HRTV for 30 min at 4°C and then for 2 hours at 37°C (**G**). The numbers of fluorescent R18 puncta were quantified by fluorescence microscopy (**H**). *P* values were determined by ordinary one-way ANOVA with Dunnett’s multiple comparison test (n  =  10) ****, P < 0.0001.

Infection study indicated that SPT-KO and UGCG-KO cells showed no detectable defects in the cell surface binding (4°C) or internalization (37°C) of HRTV (Fig 6A). To test whether GlcCer deficiency affected the intracellular trafficking of HRTV, infected cells were collected and the post-nuclear supernatants (PNS) containing organelles was subjected to fractionation *via* iodixanol density gradient centrifugation. Then, gradient fractions were analyzed by immunoblotting with the late endosome/lysosome marker LAMP1 and cytosol marker β-actin (Fig 6B). This showed that while total amounts of HRTV N protein in the PNS were comparable between control cells and UGCG-KO cells, it was detected in the late endosome/lysosome (Fractions 6–7) and cytosol (Fraction 8) in control cells, but only in the late endosome/lysosome in UGCG-KO cells (Fig 6B). In a parallel study, the distribution of HRTV S- and M-RNA from infected control cells or UGCG-KO cells was consistent with that of HRTV N protein (Fig 6B and 6C). Furthermore, electron microscopy analysis showed that a majority of HRTV particles were trapped in the endosomes of UGCG-KO cells, whereas only a few viral particles were detected in the endosomes of control cells (Fig 6D). These results suggest that GlcCer is critical for the endosome-to-cytosol trafficking of HRTV.

To further test the critical role of GlcCer in HRTV-induced membrane fusion, we labeled and purified HRTV particles with two lipophilic probes, DiOC18 and R18. Prior to membrane fusion, the green fluorescence of DiOC18 is suppressed by self-quenching and fluorescent resonance energy transfer (FRET) to R18 in the viral membrane, whereas the red fluorescence from R18 is partly self-quenched. Upon membrane fusion, removal of the self-quenching and FRET dramatically increases the green fluorescence of DiOC18. 293T cells were incubated with DiOC18/R18-labeled HRTV at 4°C for 30 mins and warmed up to 37°C for 90 mins. Extensive co-localization of R18 and DiOC18, indicating membrane fusion, was detected in control cells and UGT8-KO cells (Fig 6E and 6F). In striking contrast, the numbers of DiOC18 green fluorescence dots were markedly lower in SPT-KO and UGCG-KO cells than in control cells (Fig 6E and 6F). In addition, nitrobenzoxadiazole (NBD)-labeled green fluorescent GlcCer or GalCer was added to culture medium at 24 hours prior to infection with red fluorescent R18-labeled HRTV where R18 fluorescence is self-quenched before membrane fusion. A considerable increase of R18 red fluorescent dots in SPT-KO cells upon NBD-GlcCer supplement than upon DMSO or NBD-GalCer supplement (Fig 6G and 6H). These results demonstrate a critical role of GlcCer in HRTV-induced membrane fusion.

### HRTV or DBV glycoproteins are sufficient for GlcCer-mediated membrane fusion

**Fig 7:**
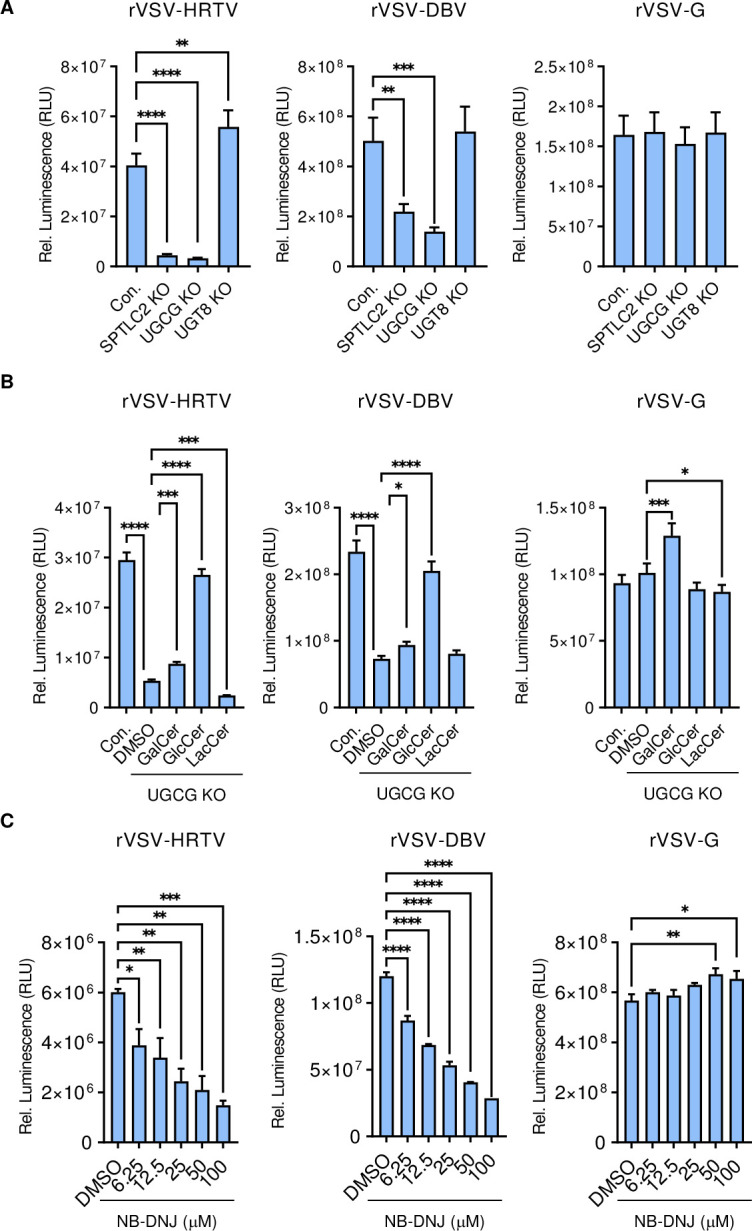
HRTV glycoproteins are responsible for GlcCer-mediated membrane fusion. **A.** Effect of SPTLC2-, UGCG- or UGT8-deficiency on rVSV-HRTV, rVSV-DBV, and rVSV-G entry. Control and KO 293T cells were infected with the indicated virus for 24 hours, followed by luciferase assay. Luminescence readings were normalized to the respective cell numbers. **B.** Rescue of rVSV-HRTV and rVSV-DBV entry in UGCG KO cells. Control and UGCG-KO 293T cells were cultured in media supplemented with 50 μM indicated glycosphingolipid for 24 hours, followed by infection with the indicated virus for additional 24 hours for luciferase assay. **C.** Effect of NB-DNJ treatment on rVSV-HRTV, rVSV-DBV, and rVSV-G entry. 293T cells were treated with indicated concentrations of NB-DNJ for 24 hours, followed by infection with the indicated virus for 24 hours prior to luciferase assay. All the data shown are means ± SEM from representative experiments (n = 3 technical replicates). *P* values were determined by ordinary one-way ANOVA with Dunnett’s multiple comparison tests. ****, *P* < 0.0001; ***, *P* < 0.001; **, *P* < 0.01; *, *P* < 0.05.

The M segment of HRTV encodes two glycoproteins, Gn and Gc, responsible for viral entry and membrane fusion. To investigate whether HRTV or DBV Gn and Gc glycoproteins were sufficient for GlcCer-mediated membrane fusion, replication-defective recombinant rVSV carrying firefly luciferase gene as a surrogate marker for infection was pseudotyped with HRTV glycoprotein (rVSV-HRTV) or DBV glycoprotein (rVSV-DBV). Luciferase assay showed that the entry of rVSV-HRTV or rVSV-DBV were drastically reduced in SPT-KO or UGCG-KO cells compared to control or UGT8 KO cells (Fig 7A). In addition, supplement of GlcCer, but not GalCer or LacCer, in UGCG-KO cells recovered the entry of rVSV-HRTV and rVSV-DBV (Fig 7B). On the other hand, neither deficiency nor supplement of GlcCer affected the entry of rVSV-G, carrying its own glycoprotein (Fig 7A and 7B). Furthermore, the UGCG inhibitor NB-DNJ inhibited rVSV-HRTV and rVSV-DBV entry, but not rVSV-G entry, in a dose-dependent manner (Fig 7C).

**Fig 8:**
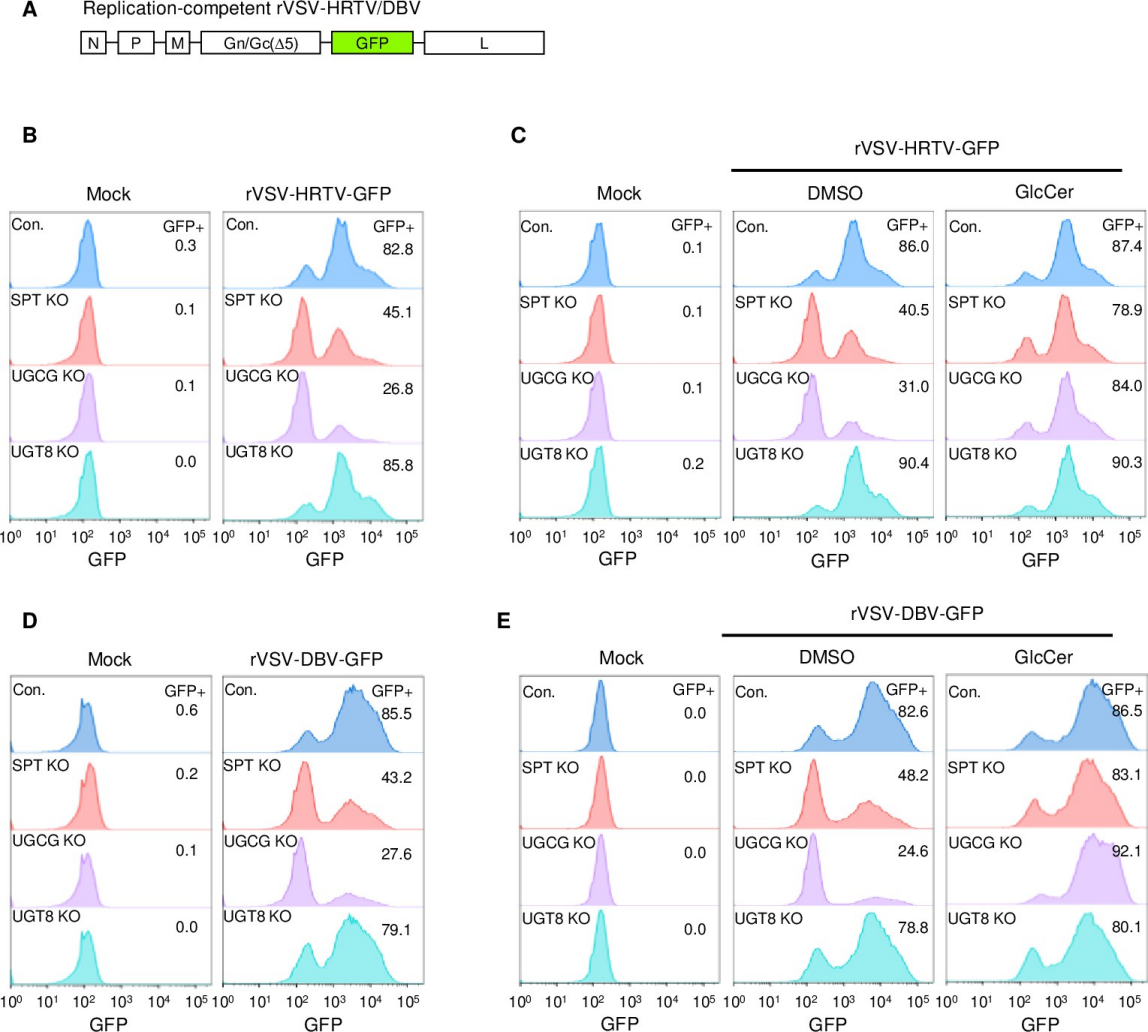
Replication-competent HRTV or DBV pseudotyped viruses require GlcCer for efficient entry. **A.** Diagram of replication-competent rVSV-DBV genome. **B.** Replication-competent rVSV-HRTV entry in SPTLC2-, UGCG- or UGT8-deficiency cells. Control and KO 293T cells were infected with replication-competent rVSV-HRTV for 16 hours, followed by flow cytometry. **C.** Rescue of replication-competent rVSV-HRTV entry in SPTLC2-, UGCG- or UGT8-deficiency cells. Control and UGCG-KO 293T cells were cultured in media supplemented with 50 μM DMSO or GlcCer for 24 hours, followed by infection with replication-competent rVSV-HRTV for 16 hours prior to flow cytometry assay. **D.** Replication-competent rVSV-DBV entry in SPTLC2-, UGCG- or UGT8-deficiency cells. **E.** Rescue of replication-competent rVSV-DBV entry in SPTLC2-, UGCG- or UGT8-deficiency cells. The experiments were performed similarly as described above.

We also generated the replication-competent rVSV-HRTV and rVSV- DBV, where the VSV G gene was replaced by the HRTV or DBV Gn/Gc along with green fluorescent protein (GFP) gene (Fig 8A). Flow cytometry analysis showed that the infectivity of replication-competent rVSV-HRTV and rVSV-DBV was significantly reduced in SPT-KO or UGCG-KO cells (Fig 8B and 8D). GlcCer supplement in SPT-KO or UGCG-KO cells robustly facilitated viral infection to the similar levels of viral infection in control or UGT8-KO cells (Fig 8C and 8E). Taken together, these results indicate that HRTV or DBV Gn/Gc proteins are sufficient for GlcCer-mediated entry.

### HRTV Gc protein is associated with GlcCer in the lipid bilayer

Low pH induces a conformational change of DBV and HRTV Gc protein to allow membrane fusion [31]. To investigate whether GlcCer affects the pH level in lysosomes, we treated control, SPT-KO, UGCG-KO, or UGT8-KO cells with pH-sensitive pHrodo dextran dye that shows low fluorescence at neutral pH and high fluorescence at low pH after endocytosis. This showed that pH values were comparable among control, SPT-KO, UGCG-KO, and UGT8-KO cells (S7A Fig). Exogenous supplement of GalCer, GlcCer or LacCer did not change the pH values of SPT-KO cells (S7B Fig).

**Fig 9:**
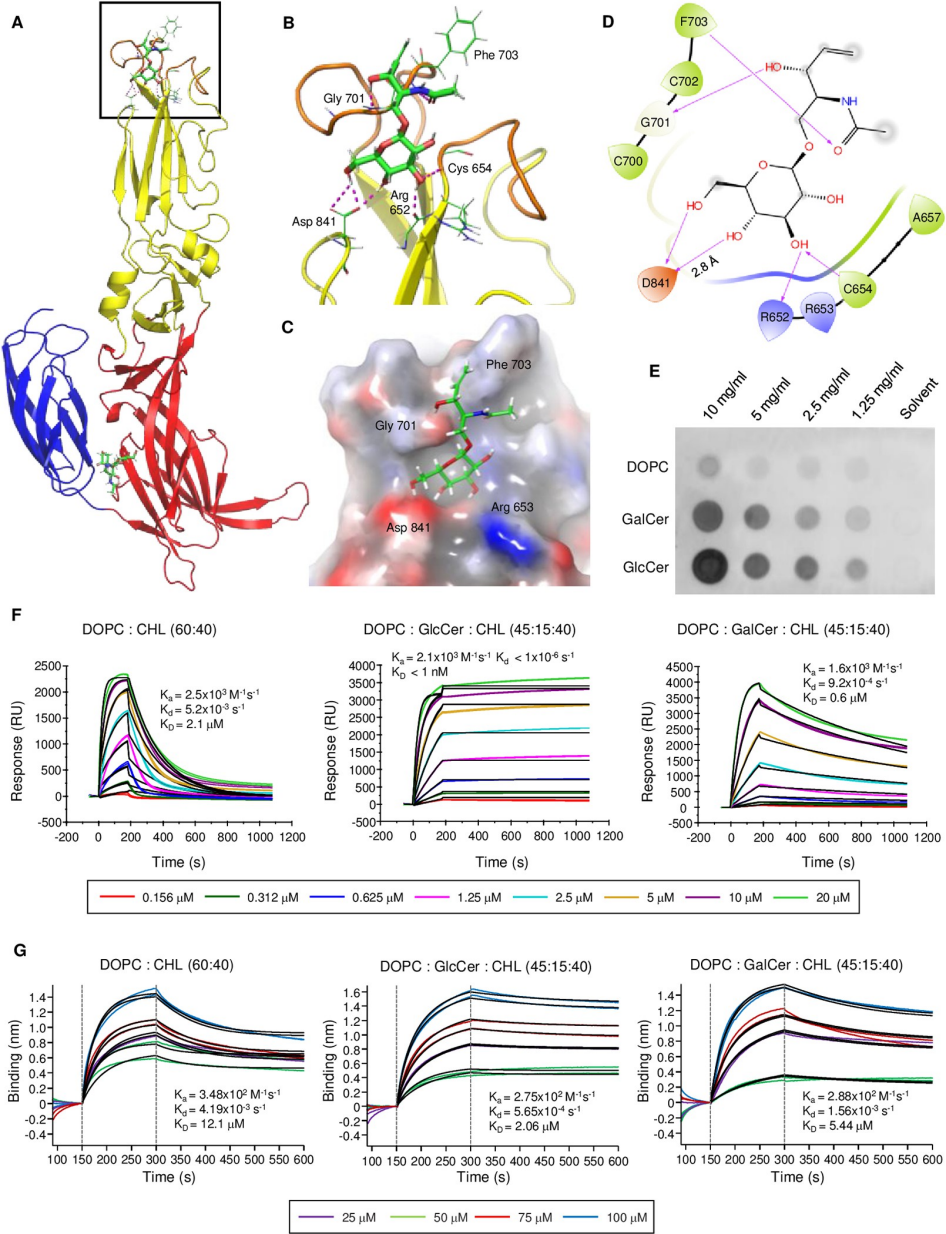
The HRTV Gc protein is associated with GlcCer in the lipid bilayer. **A.** Induced-fit docking pose of GlcCer with HRTV Gc protein. HRTV Gc ectodomain (PDB: 5YOW) is represented as ribbons and colored by domains (red, domain I; yellow, domain II; blue, domain III). The fusion loops at the tip of domain II are displayed in orange. The bound head group of GlcCer (framed) is drawn as sticks and colored according to atom type (carbon, green; nitrogen, blue; oxygen, red). **B.** Interaction of GlcCer with HRTV Gc protein in the lipid-head-group binding pocket. The hydrogen bonds between GlcCer and Gc protein are represented as dashed lines and colored in magenta. **C.** GlcCer binding groove. The HRTV Gc lipid-head-group binding pocket is shown in surface representation and colored according to electrostatic potential. **D.** 2D representation of docking interaction of GlcCer with binding site residues of HRTV Gc. The hydrogen bonds are shown in magenta lines. **E.** HRTV Gc protein directly binds to DOPC, GalCer, and GlcCer. Serially diluted DOPC, GalCer, and GlcCer were spotted onto the PVDF membrane. Protein-lipid overlay assay was performed with an anti-6xHis antibody. **F.** SPR assay of HRTV Gc binding kinetics to the indicated liposomes at neutral pH. All experiments were performed as duplicates, and the calculated fit is shown as a black line. Equilibrium constants (K_D_) are determined by measuring the association rate constant (K_a_) and dissociation rate constant (K_d_). **G.** Binding kinetics of HRTV Gc to the indicated liposomes at acidic pH. Sensorgrams measured in replicates by bio-layer interferometry (BLI) and fitting (black) of the interaction of Gc to the indicated liposomes.

Gc proteins of the *Bandavirus* genus are class II membrane fusion proteins that expose the nonpolar segmented fusion loops after the low pH-induced conformational change. The fusion loop inserts into the target membrane, which is essential for the initiation of membrane fusion. HRTV, DBV, and RVFV contain two conserved fusion loops in the domain II of their Gc proteins (S8A and S8B Fig). It has been reported that the RVFV Gc has a binding pocket for glycerophospholipid (GPL) head groups near the fusion loops [32]. Interestingly, HRTV Gc also has a similar lipid-head-group binding pocket at the tip of domain II surrounded by the conserved fusion loops (Fig 9A). To test if HRTV Gc interacted with the GlcCer head group to form a stable protein-lipid membrane complex, HRTV Gc protein and an individual head group of phosphatidylcholines (PC), GlcCer or GalCer were subjected to induced fit docking (IFD) program. This showed that the head group of PC could insert into the binding pocket of HRTV Gc as shown with RVFV Gc (S9A and S9B Fig). In addition, HRTV Gc also appears to bind to the head groups of GlcCer and GalCer (Figs 9B, 9C, S9C and S9D). Specifically, GlcCer may interact with the main chains of R652 and C654 at the fusion loop 1, the main chains of G701 and F703 at the fusion loop 2, and the side chain of D841 at the binding pocket of Gc protein (Fig 9D). To investigate potential direct binding of HRTV Gc to GlcCer, we performed protein-lipid overlay (PLO) assay. Polyvinylidene difluoride (PVDF) membrane spotted with serial dilutions of dioleoyl phosphatidylcholine (DOPC), GlcCer or GalCer standards were incubated with 6xHis-tag recombinant ectodomain of HRTV Gc (residues 567–999) purified *via* baculovirus expression (S10A and S10B Fig), followed by immunoblotting with an anti-6xHis tag antibody. PLO assay showed that Gc protein directly bound to DOPC, GalCer or GlcCer in a dose-dependent manner and the binding ability of Gc was much more robust to GlcCer than to GalCer or DOPC (Fig 9E).

To test whether GlcCer affected Gc protein insertion into the lipid bilayer, three liposomes containing DOPC, DOPC+GlcCer (3:1 ratio) or DOPC+GalCer (3:1 ratio) were generated together with 40% cholesterol (CHL) to maintain the space between lipid head groups as well as the fluidity of liposome membrane. All liposomes had the same diameter of approximate 100nm (S10C Fig). Subsequently, we measured the binding kinetics between Gc and different liposomes by surface plasmon resonance (SPR) at neutral pH (Fig 9F). SPR assay indicated that while the interaction kinetics between Gc and individual liposomes were similar at the association phase, the dissociation rate of Gc-GalCer liposome complex was slower than that of Gc-DOPC liposome complex (Fig 9F). In striking contrast, little to no dissociation was observed in the Gc-GlcCer liposome complex. In addition, we also performed bio-layer interferometry (BLI) assay at acidic pH that mimics a lysosomal environment (Fig 9G). HRTV Gc binding affinity to GlcCer liposome was much stronger than its binding affinity to GalCer or DOPC liposome. In conclusion, HRTV Gc binds to GlcCer in the lipid bilayer to permit a stable protein-membrane complex formation and lipid bilayer insertion for membrane fusion.

### The D841 residue at the Gc lipid-binding pocket is essential for GlcCer-mediated viral entry

**Fig 10:**
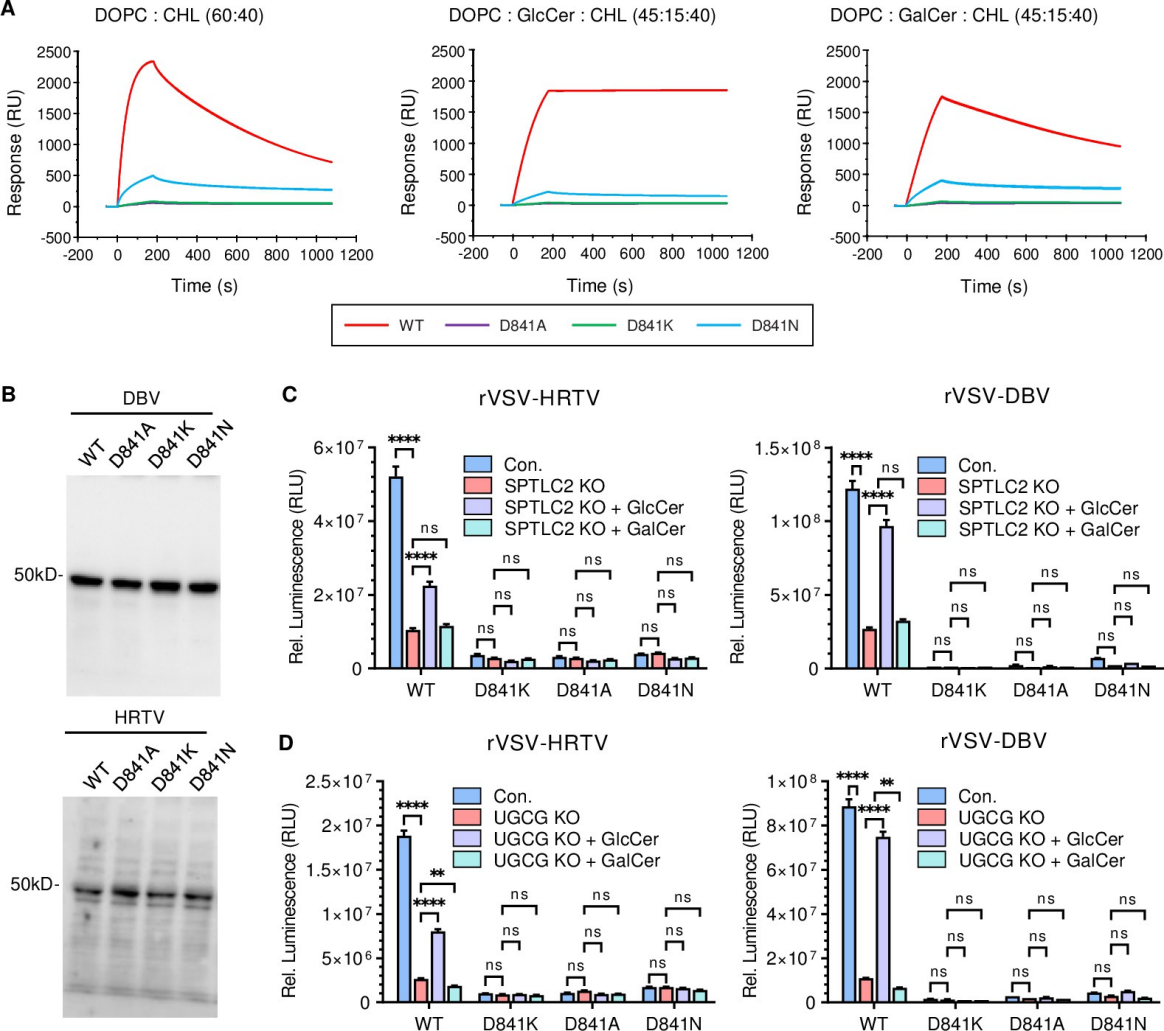
The D841 residue of Gc protein is crucial for its binding to lipid head group. **A.** SPR sensorgrams showing the interaction of Gc WT, D841A, D841K, or D841N mutant with indicated liposomes. WT and mutant Gc proteins were immobilized on the chip surface at the same response units, and the liposome (2.5 μM) was then injected over the sensor chip. **B.** Expression of WT and mutant glycoproteins of DBV and HRTV. DBV or HRTV glycoprotein expression vectors were transfected into 293T cells for 24 hours and Gc expression was analyzed by immunoblots. **C-D.** Entry efficiency of D841 mutant-containing rVSV-HRTV or rVSV-DBV in SPTLC2- and UGCG-KO cells. SPTLC2-KO and UGCG-KO 293T cells were cultured in media supplemented with 50 μM GalCer or GlcCer for 24 hours before viral infection. Luciferase activity was measured at 18 hours post-infection. Control cells without glycosphingolipids supplement was included as comparison. Data shown are means ± SEM from representative experiments (n = 3 technical replicates). *P* values were determined by two-way ANOVA with Dunnett’s multiple comparison tests. ****, *P* < 0.0001; ***, *P* < 0.001; **, *P* < 0.01; *, *P* < 0.05.

Molecular docking modeling suggested that DOPC, GlcCer, and GalCer were associated with the negatively charged D841 residue of HRTV at the Gc lipid binding pocket that is completely conserved among HRTV, DBV and RVFV. To investigate whether the D841 residue was required for membrane insertion, three mutant HRTV Gc proteins, carrying a positively charged lysine replacement (D841K), a nonpolar alanine replacement (D841A), or a polar uncharged replacement (D841N), were purified from baculovirus expression. All three proteins were eluted at the same fraction from size exclusion chromatography (S11A Fig). SPR assay showed that all three mutant proteins showed the loss of liposome binding activity though the D841N protein exhibited a minimal liposome binding activity (Fig 10A), indicating that the D841 residue of Gc protein is crucial for its binding to the lipid head group.

To demonstrate whether the binding between Gc and lipid bilayer was essential for viral entry, rVSV-HRTV or rVSV-DBV pseudotyped with wild-type (WT) or mutant Gc was constructed (Fig 10B). Compared to rVSV-HRTV-WT Gc and rVSV-DBV-WT Gc, D841K-, D841A- or D841N-containing rVSV-HRTV/DBV showed dramatic reduction of infectivity (Fig 10C and 10D). Finally, exogenous GlcCer supplemention to SPT-KO or UGCG-KO cells did not rescue the infectivity of D841K-, D841A-, or D841N-containing rVSV-HRTV/DBV (Fig 10C and 10D). These results indicate that the D841 residue at the Gc lipid-binding pocket is essential for Gc-mediated insertion and viral entry.

## Discussion

HRTV is a recently identified emerging bandavirus causing tick-borne viral disease (TBVD) in the United States. There is an urgent need to understand the interplay between HRTV and its host, which can facilitate the development of antiviral drugs and treatment approaches. To identify host factors for HRTV infection, we performed a CRISPR/Cas9 knockout screen and identified that the glycosphingolipid synthesis pathway is required for HRTV infection. HRTV replication was significantly reduced upon treatment with NB-DNJ (also known as Miglustat and Zavesca), which is a specific pharmacological inhibitor for UGCG. NB-DNJ was initially developed as a potential antiviral drug for HIV and is currently a US FDA-approved drug for Gaucher disease caused by the accumulation of GlcCer in lysosomes [33]. Our data suggest that NB-DNJ has the potential to restrict viral transmission and prevents the establishment of productive infections upon HRTV and DBV infections. However, the safe and effective use of NB-DNJ in treating HRTV or DBV infections requires further investigation.

The glycosphingolipids consist of glycans conjugated to a ceramide core and comprise over 300 different complex molecules based on variation in the glycan assembly [34]. In the glycosphingolipid metabolic pathway, ceramide, the common substrate of UGCG and UGT8, occupies a central position. While UGT8 was enriched from CRISPR screen, its rank was not as high as that of other genes. Moreover, we did not observe any detectable change of viral entry in UGT8-KO cells. This suggests that extended period of the CRISPR screen, including three rounds of HRTV infection and cell death, might have led to the enrichment of host factors that play minor roles in viral entry. Our CRISPR/Cas9 knockout showed that deficiency of SPTLC2 or UGCG upstream of GlcCer synthesis markedly inhibited HRTV and DBV infection, whereas deficiency of B4GALT5/6 synthases downstream of GlcCer synthesis enhanced HRTV and DBV infection. In addition, exogenous GlcCer supplement into culture medium of SPTLC2 or UGCG knock-out cells was able to rescue HRTV and DBV infectivity. These results clearly demonstrate the critical role of GlcCer in the infectivity of HRTV and DBV. It should be noted that despite the strong knockout efficiency of SPTLC2 in both cells, SPTLC2-KO 293T cells still exhibited low SPT enzymatic activity. One possible explanation is that the serine palmitoyltransferase (SPT) complex comprises of SPTLC1 and SPTLC2 or SPTLC3 along with either SPTSSA or SPTSSB, and that SPTLC2 and SPTLC3 are functionally complementary [35]. Thus, SPTLC2 knockout alone might lead to partial loss of SPT activity in 293T cells due to the presence of intact SPTLC3 expression.

*Bandavirus* infection is a multi-step process including attachment, internalization, trafficking from early endosome to lysosome, membrane fusion, and genome release into the cytoplasm [36]. Our data indicate that while GlcCer-deficiency does not impair the binding or internalization of HRTV, it blocks the membrane fusion step of HRTV in the lysosome. These results are consistent with a previous study of DBV entry [29]. Furthermore, the study with two DiOC18 and R18 lipophilic probe-labeled HRTV particles showed that HRTV-induced membrane fusion was dramatically reduced in GlcCer deficient cells. Finally, the entry of rVSV-HRTV or rVSV-DBV were drastically reduced in SPT-KO or UGCG-KO cells compared to control or UGT8 KO cells. Taken together, previous [29] and our study unambiguously demonstrate that GlcCer plays a critical role in the membrane fusion step of HRTV and DBV.

In neutral pH, Gc is in the pre-fusion state and forms a heterodimer with Gn on the virion surface. Its proton binding in the lysosome triggers Gn/Gc dissociation and exposure of the fusion loops at the tip of Gc [37]. Then, the fully extended Gc inserts its fusion loops into the endosomal membrane, forming a trimeric post-fusion conformation on the virion surface [38]. Lipid bilayer fusion requires stable insertion to overcome a high energy barrier. The interaction between the Gc and the insertion site must withstand the pulling forces when driving the merging of viral envelope and target lysosomal membrane [39]. Interestingly, RVFV Gc contains a glycerophospholipid-specific pocket that drives target membrane insertion [32]. Similarly, GlcCer provides a stable binding anchor for HRTV and DBV Gc insertion into the lysosomal membrane. PLO assay showed strong GlcCer binding activity of HRTV Gc and IFD revealed a GlcCer lipid-head-group binding pocket of HRTV Gc. Finally, SPR and BLI assays confirmed that Gc insertion into the GlcCer-containing lipid bilayer was significantly more stable than its insertion into GalCer-containing or DOPC lipid bilayer at either neutral or acidic pH. Thus, HRTV and DBV Gc binds to GlcCer in the lipid bilayer to permit a stable protein-membrane complex formation and lipid bilayer insertion for membrane fusion.

GlcCer and GalCer consist of D-glucose and D-galactose residues linked to ceramide, respectively. They represent almost identical structures since D-glucose and D-galactose are epimeric at the carbon-4 (C4) position [40] except that the hydroxyl groups in GlcCer and GalCer are oriented differently at this position, altering their physicochemical property. In the docking results, there is a hydrogen bond between the D841 of HRTV Gc and the hydroxyl group of the C4 position of GlcCer and GalCer. The different lengths and angles of hydrogen bonds formed between HRTV Gc and GlcCer or GalCer may cause differences in their binding affinity. In addition, the D841 residue at the Gc lipid-head-group binding pocket is also associated with the positively charged trimethylamine group of DOPC that is the most abundant phospholipid in mammalian cells. The initial insertion is most likely mediated by nonspecific hydrophobic interactions *via* the fusion loop of Gc protein, resulting in virion enrichment at the surface of endosomal membrane. Subsequently, Gc binds to GlcCer to form a stable protein-lipid complex, which facilitates efficient membrane fusion. The fact that D841 mutations abolish the binding between Gc and liposomes further supports essential role of the D841 residue for stable Gc-membrane complex formation.

In conclusion, our results indicate that after HRTV and DBV internalization, GlcCer in the endosomal membrane associates with Gc protein as an intracellular receptor (S12 Fig). The stable interaction between Gc and GlcCer is required for efficient viral membrane fusion. Our findings reveal a novel mechanism of how the sphingolipid metabolism pathway regulates the entry of HRTV and DBV, which ultimately provides fundamental insights for drug or vaccine development against the viral infection.

## Materials and methods

### Cells, viruses, antibodies, and reagents

HEK293T and HeLa cells were purchased from ATCC. All the cells were cultured in Dulbecco’s modified Eagle’s medium (DMEM; Gibco) supplemented with 10% fetal bovine serum (FBS; Gibco) at 37°C with 5% CO_2_. The Sf9 insect cells were cultured in ExpiSf CD medium (Gibco, A37678-02), and the Tni insect cells were cultured in ESF 921 (Expression systems, 96-001-01). Heartland bandavirus (Strain: MO4) is from BEI.

Mouse antibodies against Flag (Sigma-Aldrich, F1804), 6xHis (BioLegend, 906102), LAMP1 (Santa Cruz, sc-20011), β-actin (Santa Cruz, sc-47778) were purchased for the indicated manufacturers. The monoclonal antibody (2AG8) against the HRTV N protein is from CDC [41].

NB-DNJ (Caymanchem, 21065), UGT8 Inhibitor 19 (Caymanchem, 32723), GalCer (Avanti polar lipids, 860521), GlcCer (Avanti polar lipids, 860539), LacCer (Matreya, 1507), OptiPrep density gradient medium (Sigma-Aldrich, D1556), DiOC18 (Thermofisher, D275), R18 (Thermofisher, O246), NBD-GlcCer (Matreya, 1622–001), NBD-GalCer (Matreya, 1633–001), DOPC (Avanti polar lipids, 850375), cholesterol (Avanti polar lipids, 700000), pHrodo green dextran (Invitrogen, P35368) were purchased from the indicated manufacturers.

### Genome-wide CRISPR knockout library screen

The human CRISPR knockout pooled library A (GeCKO v2A) that contains 65,383 sgRNA and targets 19,050 human genes was a gift from Feng Zhang (Addgene #1000000049)[30]. The workflow of this genetic screen is illustrated in Fig 1A. First, we generated a stable Cas9-expressing 293T cell line (293T-Cas9) by lentiviral transduction of lentiCas9-Blast plasmid. Then 1 x10^8^ 293T-Cas9 cells were transduced with the GeCKO v2A library at an MOI of 0.3 to ensure that most cells received only one sgRNA. Cells were selected with 1 μg/ml puromycin for 10 days and then pooled together. Approximately 6 x 10^7^ mutagenized cells were infected with HRTV MO4 strain at MOI of 1 and then incubated until nearly all cells were killed. The surviving cells were then harvested and reseeded to the flasks. After the additional two rounds of HRTV challenge, the survival cells were expanded, and 3 × 10^7^ cells were collected for genomic DNA extraction and PCR amplification. Genomic DNA from the uninfected cells (3 ×10^7^) was extracted as the control. The amplified sgRNA sequences were subjected to next-generation sequencing using an Illumina platform. The gene enrichment was analyzed by MAGeCK. The Kyoto Encyclopedia of Genes and Genomes (KEGG) analysis was performed by DAVID (https://david.ncifcrf.gov/).

### qRT-PCR

Total RNA from cells was extracted with the TRI reagent (Sigma-Aldrich, T9424). The cDNA was synthesized with the iScript reverse transcription supermix (Bio-Rad, 1708840). Viral or host RNA levels were determined using the SsoAdvanced universal SYBR green supermix (Bio-Rad, 172–5270) on CFX Connect Real-Time System (Bio-Rad) instrument with GAPDH as the endogenous reference. The primers used are as follows:

GAPDH-Fwd: 5’-GTCTCCTCTGACTTCAACAGCG-3’;

GAPDH -Rev: 5’-ACCACCCTGTTGCTGTAGCCAA-3’;

SPTLC2-Fwd: 5’-CCAGACTGTCAGGAGCAACCAT-3’;

SPTLC2-Rev: 5’-TTCGTGTCCGAGGCTGACCATA-3’;

UGCG-Fwd: 5’-CTTGGTTCACGGGCTGCCTTAC-3’;

UGCG-Rev: 5’-GAAACCAGTTACATTGGCAGAGAT-3’;

UGT8-Fwd: 5’-GGAGGAATCCTAACCAAACCAGC-3’;

UGT8-Rev: 5’-TCCTGCCAGTTTGTTAGCAATGTC-3’;

B4GALT5-Fwd: 5’-GAAGATGACGACCTCTGGAACAG-3’;

B4GALT5-Rev: 5’-GCCGTTCTTTTGACTTCCTCAGC-3’;

B4GALT6-Fwd: 5’-CTCATTCCTTTCCGTAATCGCCA-3’;

B4GALT6-Rev: 5’-GCCCACATTGAAAAGCATCGCAC-3’;

HRTV S segment-Fwd: 5’-GAGAGACGTAGGCGAATGGG-3’;

HRTV S segment-Rev: 5’-GTGAGACCCCTAACACGCAA-3’;

HRTV M segment-Fwd: 5’-CTAAGCCACTCAGGCACATAT-3’;

HRTV M segment-Rev: 5’-GTCCCTCTCTGACAGACTACTT-3’;

### 50% Tissue Culture Infectious Dose (TCID50) assays

The indicated cells were seeded at a density of 5000 cells per well in 96-well plate the day before infection. Half-log serial dilutions of HRTV or rVSV-DBV were then added to four replicate wells and incubated for 72 hours. Following incubation, CellTiter-Glo (Promega) reagent was added to each well and luminescence was measured. The TCID_50_ value was calculated as the reciprocal of the dilution that resulted in a 50% reduction in ATP levels compared to untreated controls. The data was plotted using GraphPad Prism and analyzed by a dose-response (variable slope) algorithm to determine the TCID_50_ value.

### SFC-MS/MS analysis of GlcCer and GalCer

For each sample, 1 x 10^7^ cells were collected and sent to Creative Proteomics for quantitative analysis of GlcCer and GalCer using an SFC-MS/MS method.

### Immunofluorescence microscopy

Virus-infected cells were washed twice with PBS and fixed with 4% paraformaldehyde (PFA) in PBS for 30 min at room temperature. Next, the cells were permeablized with 0.2% Triton X-100 for 1 h and washed three times with PBS. After blocking with PBS- 3% BSA for 1 hour, cells were incubated with mouse monoclonal antibody (2AG8) against the HRTV N protein at 4°C overnight. After three additional washes in PBS, cells were incubated with the goat anti-mouse secondary antibody conjugated with Alexa Fluor 594 (Invitrogen #A-11005) for 1 h at room temperature. The nuclei were stained with DAPI for 2 min and then washed with PBS for 3 times. Images were collected using Keyence BZ-X 810 fluorescence microscope.

### Viral binding and internalization assay

Control and KO HeLa cells were seeded in 12-well plate one day before the assays. Cells were pre-incubated on ice for 10 minutes to inhibit endocytosis and then incubated with HRTV (MOI = 10) in cold medium at 4°C for 1 hour. After five times washing with ice-cold PBS, cells were lysed in TRI reagent for RNA extraction to determine the viral binding on the cell surface. For internalization assay, after PBS washing, cells were moved to 37°C in DMEM with 2% FBS. After 1 hour of incubation, cells were washed three times and trypsinized for 10 minutes to remove surface-bound viruses. After three additional washes in PBS, the cells were collected, and total RNA was extracted for qPCR assay.

### Subcellular fractionation

The protocol for the subcellular fractionation method was described previously [42]. Briefly, control and UGCG-KO HeLa cells were seeded in T175 flasks one day before infection. Cells were incubated with HRTV (MOI = 10) at 4°C for 1 hour. Then the cells were washed with PBS and incubated at 37°C in DMEM with 2% FBS for additional 2 hours. The cells were harvested and resuspended in 800 μL homogenization buffer (HB) with protease inhibitors and homogenized using a Dounce homogenizer. After centrifugation at 1000 x g for 10 minutes, the supernatant [postnuclear supernatant (PNS)] was adjusted to a concentration of 30% OptiPrep solution. 1 mL diluted PNS solution was transferred to an ultracentrifuge tube and overlayed with 1 mL of 20%, 1 mL of 15%, 1 mL of 10%, 0.5 mL of 5% OptiPrep solutions, and then 0.5 mL of HB on the top. After centrifugation at 100,000 × g for 16 h at 4°C, the fractions (F1-F10) were collected from top to bottom. The samples from each fraction were used for Western blotting analysis by indicated antibodies. The viral RNA from each fraction was extracted by the QIAamp viral RNA mini kit (QIAGEN, 52904) and analyzed by qPCR.

### Transmission electron microscope

Control and UGCG-KO 293T cells cultured in a 6-well plate were incubated with HRTV (MOI = 50) at 4°C for 30 minutes and then at 37°C for 2 hours. Cells were harvested by cell scraper and fixed with 2.5% glutaraldehyde and 2% PFA. The representative images were collected by FEI Tecnai G2 Spirit BioTWIN transmission electron microscope.

### Membrane fusion assay

The membrane fusion assay with 3,3’-dioctadecyloxacarbocyanine perchlorate (DiOC18) and Octadecyl Rhodamine B chloride (R18) labeled virus was described previously [43, 44]. Briefly, to generate DiOC18 and R18 double labeled HRTV, purified virus (viral protein, 100 μg) was incubated with DiOC18 (200 nM) and R18 (400 nM) in 1 mL PBS for 1 hour at room temperature. To generate R18 labeled HRTV, the purified virus (viral protein, 100 μg) was incubated with R18 (2 μM) in 1 mL PBS for 1 hour at room temperature. After labeling, the unbound probe was removed with pierce dye removal columns (Thermofisher, 24020117). The labeled virus was passed through a 0.22 μm filter (GenClone, 25–243) and stored at 4°C before infection.

### Generation of pseudotyped viruses

293T cells were transfected with pCAGGS-HRTV-GP, pCAGGS-DBV-GP, or pMD2. G. 24 hours after transfection, cells were infected with G-complemented VSVΔG/Luc at MOI of 0.5. 2 hours after infection, cells were washed with PBS for three times to remove unbound virus. The supernatant containing pseudotyped virus was collected 24 hours after infection and then clarified by spinning at 4000 r.p.m. for 15 min. After centrifugation, the supernatant was aliquoted and stored at −80°C for use.

### Generation of replication-competent rVSV-HRTV/DBV

The protocol for recovery of VSV pseudotypes containing the envelope glycoproteins of heterologous viruses from plasmids was described previously [45]. Briefly, the VSV-ΔG-GFP-2.6 plasmid expression vector system was purchased from kerafast (EH1027). The codon-optimized HRTV or DBV glycoprotein with deletion of last 5 amino acids was cloned into the pVSV-delta-G-GFP-2.6 to construct the pVSV-HRTV/DBV-GFP plasmid. 293T cells were co-transfected with pVSV-HRTV/DBV-GFP, pCAGGS-T7 polymerase, and helper plasmids encoding VSV N, P, G, and L protein in a ratio of 5:5:3:5:3:1 in 6-well plate. The total amount of DNA mixture used for one well was 10 μg. The virus recovery supernatant was harvested 72 hours post-transfection and inoculated to Vero-E6 cells. After infection, the supernatant was harvested when 90% of the cells showed signs of VSV-induced CPE. Followed by two times passages, the supernatant containing replication-competent rVSV-HRTV or rVSV-DBV (5 x 10^6^ pfu/mL) was collected and stored at −80°C.

### Induced fit docking

The structure of HRTV Gc protein (PDB: 5YOW) was applied with the induced-fit docking (IFD) method in the Schrodinger software suite. The protein structure was prepared by Protein Preparation Wizard module. Hydrogen atoms were added to the protein structure and the missing side chains and loops were filled with Prime module. The water molecules were removed from the structure, and the protein was minimized using the OPLS4 force field. All ligands were prepared using Ligprep and optimized with the OPLS4 force field. The extended sampling protocol was performed to dock lipid-head-groups inside the ligand binding pocket of HRTV Gc protein. The diagram of the interaction between lipid and protein was visualized by 2D sketcher module. The graphical picture of resulting protein-lipid complex was made by Pymol program.

### Protein lipid overlay (PLO) assay

The protocol of protein lipid overlay assay for studying lipid-protein interaction was described previously [46]. Briefly, the DOPC, GlcCer and GalCer were dissolved in MeOH at a final concentration of 10 mg/mL. Then the lipids at different dilutions were spotted on a polyvinylidene fluoride (PVDF) membrane. The membranes are dried at room temperature for 1 hour and then incubated with 2 μg/mL recombinant 6xHis-tagged HRTV Gc protein in PBST blocking buffer (PBST containing 5% skimmed milk) at 4°C overnight. Subsequently, the membrane was washed 3 times with PBST and incubated with anti-6xHis antibody at room temperature for 1 h. Then the membrane was washed another 3 times with PBST and incubated with horseradish peroxidase (HRP)-conjugated secondary antibody at room temperature for 1 h. After additional 3 times wash with PBST, the proteins bound to the membrane are detected by enhanced chemiluminescence (ECL) detection reagents.

### Protein expression and purification

The HRTV Gc ectodomain (residues 567 to 999) and its mutations were purified by the baculovirus expression system. Specifically, a codon-optimized DNA sequence encoding the HRTV Gc ectodomain was cloned into the pFastBac1 vector in frame with an N-terminal IL-2 signal sequence and a C-terminal 6×His tag. Then the pFastBac construct was transformed into DH10Bac competent cells, and the recombinant bacmids were extracted for subsequent transfection in Sf9 insect cells with ExpiFectamine Sf transfection reagent (Gbico, A38915). The supernatant containing baculovirus was harvested at 96h after transfection and stored at 4°C. Then 1L Tni insect cells (2x10^6^ cells/mL) were infected with baculovirus at an MOI of 4 for 72 hours. The supernatant containing recombinant protein was collected for purification. The Gc protein in the supernatant was purified by high-performance immobilized metal affinity chromatography (IMAC) using a 5-mL HisTrap HP column (Cytiva, 17524802) and further purified by high-resolution size exclusion chromatography (SEC) using a HiLoad 16/600 Superdex 200 pg colum (Cytiva, 28989335) in 20 mM Tris-HCl (pH 8.0) and 50 mM NaCl. The fractions containing Gc protein were collected, and the protein was concentrated to 1mg/mL.

### Liposome preparations

The liposomes were generated using the NanoAssemblr Ignite instrument (Precision NanoSystems). The DOPC, GalCer, GlcCer and cholesterol were dissolved in ethanol at a concentration of 12.5 mM. Then one volume of lipid mixtures at the appropriate ratios and three volumes of PBS were injected into the microfluidic cartridge at a total flow rate of 12 mL/min and a flow rate ratio of 3:1. The resulting liposomes were diluted in 50ml PBS and then concentrated to 1 mL by ultrafiltration using a 10 kDa Amico ultra-15 centrifugal filter units (MilliporeSigma, UFC910096). The size and concentration of each liposome were measured by nanoparticle tracking analysis on Zetaview instrument.

### Surface plasmon resonance (SPR) analysis

The surface plasmon resonance experiments were performed using a Biacore S200 (Cytiva) equipped with a series S sensor chip NTA (Cytiva, 28994951) at a temperature of 25°C. The purified 6xHis tagged HRTV Gc protein was immobilized by metal chelation on the chip surface. Specifically, the Gc protein at a concentration of 20 μg/mL in PBS-BSA buffer (PBS containing 0.2 mg/mL BSA, pH 7.4) was immobilized at a density of 3000 RU on flow cell 2; flow cell 1 was left blank to serve as a reference surface. To collect kinetic binding data, liposomes in PBS-BSA buffer were injected over the two flow cells at different lipid concentrations at a flow rate of 5 μL/min. The complex was allowed to associate and dissociate for 300 and 900 s, respectively. The surfaces were regenerated with 1 minute injection of 350mM EDTA at a flow rate of 30 μL/min and then with 30 s injection of a mixture of 2 parts ethanol and 3 parts 50 mM NaOH at a flow rate of 10 μL/min. All the experiments were done in duplicate. The obtained data analysis was performed using Biacore S200 evaluation software with a fit model of 1:1 binding.

### Biolayer Interferometry (BLI) assay

The BLI assay was performed using the Octet N1 system (Sartorius) at room temperature. The Ni-NTA biosensor (Sartorius, 18–5101) was dipped in PBS-BSA buffer for 10 minutes before use. The binding assay protocol for each liposome included the following steps: baseline for 30 s; immobilization of 6xHis tagged HRTV Gc protein at 10 μg/mL for 60 s; baseline for 30 s; association for 150 s; and dissociation for 300 s. All the binding assays were performed in citrate buffer (0.1 M citrate buffer with 0.2 mg/mL BSA, pH5). After immobilization, the assay buffer only was used as a reference sample and subtracted from the raw data. All the experiments were done in duplicate. Data analysis was performed using Octet data analysis software with a global fit model.

### Statistical analysis

All statistical analyses were performed using Graphpad Prism 8.0. Data are shown as the mean ± SEM. The unpaired *t*-tests or ordinary one-way ANOVA with Dunnett’s multiple comparison test was used to determine statistical significance for comparison between two groups. P values of <0.05 were considered significant. *P < 0.05, **P < 0.01, ***P < 0.001 and ****P < 0.0001, ns, no significance.

## Supporting information

S1 Fig HRTV requires GlcCer for efficient infection in HeLa cell. **A.** Knockout efficiency of *SPTLC2-*, *UGCG-* or *UGT8* gene in HeLa cell lines was measured by qPCR. Data shown are means ± SEM from representative experiments (n = 3 technical replicates). *P* values were determined by multiple unpaired t-tests. **, *P* < 0.01; *, *P* < 0.05. **B.** SPTLC2 protein levels in 293T and HeLa knockout cells. **C.** Control and KO HeLa cells were infected with HRTV (MOI = 1) for the indicated times for qPCR analysis. Data shown are means ± SEM from representative experiments (n = 3 technical replicates). *P* values were determined by ordinary one-way ANOVA with Dunnett’s multiple comparison tests. ****, *P* < 0.0001; ***, *P* < 0.001; **, *P* < 0.01; *, *P* < 0.05. **D-E.** Effects of SPTLC2-, UGCG- or UGT8-deficiency on HRTV gene expression. Control or KO HeLa cells were infected with HRTV or VSV-GFP for 24 hours (MOI = 4), followed by immunostaining with an anti-N monoclonal antibody for immunofluorescence microscopy (**D**). Infectivity was quantified by ImageJ (**E**). *P* values were determined by ordinary one-way ANOVA with Dunnett’s multiple comparison test (n  =  6–11) ****, P < 0.0001.
(TIF)

S2 Fig Validation of SPTLC2 editing. **A-B.** Sanger sequencing of *SPTLC2* in control and knockout 293T or HeLa cells. Sequencing data were analyzed by ICE CRISPR Analysis Tool (https://ice.synthego.com). The guide sequences are represented by a horizontal black underlined region, the PAM sites are shown with a red underline, and the actual cut sites are indicated by a vertical black dotted line.
(TIF)

S3 Fig Validation of UGCG editing. **A-B.** Sanger sequencing of *UGCG* in control and knockout 293T or HeLa cells. Sequencing data were analyzed by ICE CRISPR Analysis Tool (https://ice.synthego.com). The guide sequences are represented by a horizontal black underlined region, the PAM sites are shown with a red underline, and the actual cut sites are indicated by a vertical black dotted line.
(TIF)

S4 Fig Validation of UGT8 editing. **A-B.** Sanger sequencing of *UGT8* in control and knockout 293T or HeLa cells. Sequencing data were analyzed by ICE CRISPR Analysis Tool (https://ice.synthego.com). The guide sequences are represented by a horizontal black underlined region, the PAM sites are shown with a red underline, and the actual cut sites are indicated by a vertical black dotted line.
(TIF)

S5 Fig Validation of B4GALT5 and B4GALT6 editing in single gene knockout cell. **A-B.** Sanger sequencing of *B4GALT5* or *B4GALT6* in control and knockout 293T cells. Sequencing data were analyzed by ICE CRISPR Analysis Tool (https://ice.synthego.com). The guide sequences are represented by a horizontal black underlined region, the PAM sites are shown with a red underline, and the actual cut sites are indicated by a vertical black dotted line.
(TIF)

S6 Fig Validation of B4GALT5 and B4GALT6 editing in double knockout cell. **A-B.** Sanger sequencing of *B4GALT5* or *B4GALT6* in control and double knockout 293T cells. Sequencing data were analyzed by ICE CRISPR Analysis Tool (https://ice.synthego.com). The guide sequences are represented by a horizontal black underlined region, the PAM sites are shown with a red underline, and the actual cut sites are indicated by a vertical black dotted line.
(TIF)

S7 Fig Deficiency of GlcCer does not change endosomal pH. **A.** Control and KO cells were incubated with pHrodo green dextran for 2 hours and fluorescence was measured with a microplate reader. **B.** SPTLC2-KO 293T cells were cultured in media supplemented with 50 μM indicated glycosphingolipids for 24 hours and then incubated with pHrodo green dextran for additional 2 hours. Fluorescence was measured with a microplate reader, and NC cells without glycosphingolipids supplement were included for comparison. Data shown are means ± SEM from representative experiments (n = 3 technical replicates). *P* values were determined by ordinary one-way ANOVA with Dunnett’s multiple comparison tests.
(TIF)

S8 Fig Comparison of Gc protein between HRTV, DBV, and RVFV. **A.** Diagram of the HRTV M segment. Gc ectodomain is colored by domains (red, domain I; yellow, domain II; blue, domain III). TM, transmembrane domain. **B.** Sequence alignment of Gc glycoproteins of HRTV, DBV, and RVFV. The amino acids interacting with GlcCer in HRTV Gc are marked with asterisks.
(TIF)

S9 Fig Induced-fit docking pose of HRTV Gc protein with head groups of DOPC and GalCer. **A.** Induced-fit docking pose of DOPC with HRTV Gc protein. The hydrogen bonds and salt bridge between DOPC and Gc are colored with magenta and cyan, respectively. **B.** 2D representation of docking interaction of GlcCer with the binding site residues of HRTV Gc. The hydrogen bonds are shown in magenta lines with arrows and the salt bridge is between residue D841 and the trimethylamine group of DOPC. **C.** Induced-fit docking pose of GalCer with HRTV Gc protein. **D.** 2D representation of docking interaction of GalCer with the binding site residues of HRTV Gc.
(TIF)

S10 Fig HRTV Gc protein purification and liposome preparation. **A.** Size exclusion chromatography (SEC) analysis of purified HRTV Gc. SEC was performed on a HiLoad 16/600 Superdex 200pg column (Cytiva) equilibrated in 20 mM Tris-HCl (pH 8.0) and 50 mM NaCl. The eluate was analyzed for absorbance at 280 nm. **B.** Coomassie blue staining of purified HRTV Gc. SDS-PAGE samples collected from SEC were stained by Coomassie blue with the molecular weight (approximately 45 kDa). **C.** Nanoparticle tracking analysis (NTA) showing the distribution of diameters and size of the indicated liposome.
(TIF)

S11 Fig Purification of HRTV mutant Gc proteins. **A.** SEC analysis of purified HRTV Gc mutant proteins. HRTV Gc WT and mutants were collected from the same fractions for subsequence binding assay.
(TIF)

S12 Fig Role of glycosphingolipid biosynthesis pathway in HRTV and DBV entry. Sphingolipid *de novo* synthesis pathway is initiated in the ER, where palmitoyl-CoA and serine are catalyzed into ceramide by enzymes, including SPT and CerS. Subsequently, ceramide is converted to GalCer by UGT8 in the ER or directly transported to the Golgi complex. In the Golgi complex, ceramide is further converted into GlcCer by UGCG. GlcCer is then converted to other complex GSLs by adding variable carbohydrate groups. GlcCer and GSLs are delivered to the plasma membrane by vesicular transport and GlcCer in the plasma membrane is subsequently recycled from cell surface and degraded in the lysosome *via* the endocytic pathway. HRTV infection is triggered by an interaction between viral glycoproteins and host receptor(s) on cell surface. Virus particles are then internalized and enter early endosomes. During viral trafficking, endosome fuses with lysosome, inducing the disassociation of Gn/Gc dimer and the conformational change of Gc protein. Subsequently, Gc protein inserts into target membrane and interacts with GlcCer to form a stable protein-lipid complex, which is essential for an efficient membrane fusion for viral genome releases its genome to the cytosol. 3-KDS, 3-ketodihydrosphingosine; Cer, ceramide; CerS, ceramide synthase; ER, endoplasmic reticulum; GalCer, galactosylceramide; GlcCer, glucosylceramide; GSL, glycosphingolipid; SPT, serine palmitoyltransferase; UGCG, ceramide Glucosyltransferase; UGT8, uridine diphosphate glycosyltransferase 8. Figure created with BioRender.com.
(TIF)

S1 File Genes identified in CRISPR/KO screen. The CRISPR/KO results included the Gene ID, number of identified sgRNA, false-discovery rate, and p-value, with the results ranked by p-value.
(XLSX)

S2 File Quantitative analysis of GlcCer and GalCer levels. The indicated samples were collected for quantitative measurement of GlcCer and GalCer, levels are quantified using an SFC-MS/MS method.
(XLSX)
