# Supplementary figures and images for: Glucosylceramide is essential for Heartland and Dabie bandavirus glycoprotein-induced membrane fusion
Source: PLoS Pathog. 2023 Mar 15;19(3):e1011232. doi: 10.1371/journal.ppat.1011232 (PMC10016662; doi:10.1371/journal.ppat.1011232)

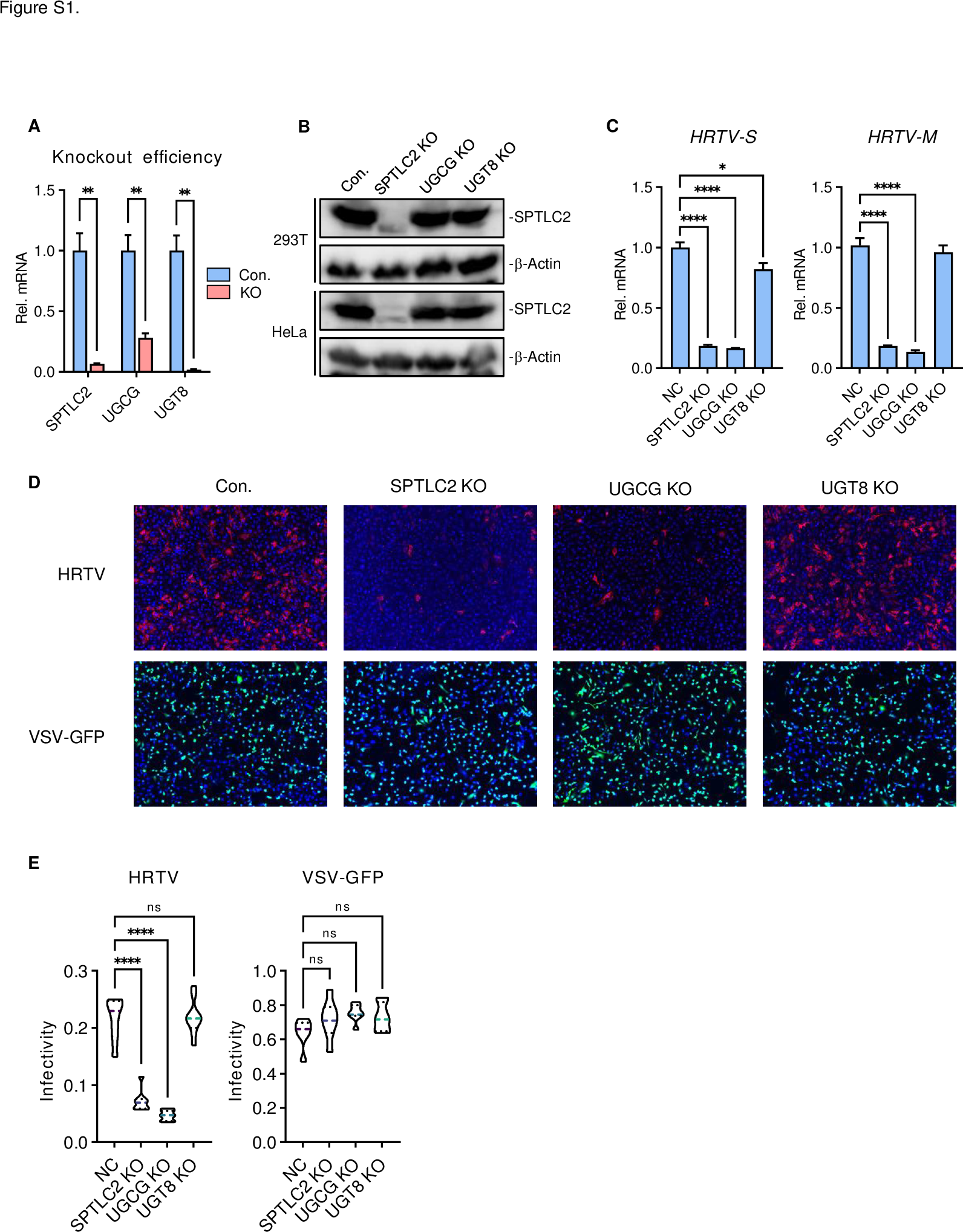

Supplement: S1 Fig — A. Knockout efficiency of SPTLC2-, UGCG- or UGT8 gene in HeLa cell lines was measured by qPCR. Data shown are means ± SEM from representative experiments (n = 3 technical replicates). P values were determined by multiple unpaired t-tests. **, P < 0.01; *, P < 0.05. B. SPTLC2 protein levels in 293T and HeLa knockout cells. C. Control and KO HeLa cells were infected with HRTV (MOI = 1) for the indicated times for qPCR analysis. Data shown are means ± SEM from representative experiments (n = 3 technical replicates). P values were determined by ordinary one-way ANOVA with Dunnett’s multiple comparison tests. ****, P < 0.0001; ***, P < 0.001; **, P < 0.01; *, P < 0.05. D-E. Effects of SPTLC2-, UGCG- or UGT8-deficiency on HRTV gene expression. Control or KO HeLa cells were infected with HRTV or VSV-GFP for 24 hours (MOI = 4), followed by immunostaining with an anti-N monoclonal antibody for immunofluorescence microscopy (D). Infectivity was quantified by ImageJ (E). P values were determined by ordinary one-way ANOVA with Dunnett’s multiple comparison test (n = 6–11) ****, P < 0.0001. (TIF) [file ppat.1011232.s001.tif]

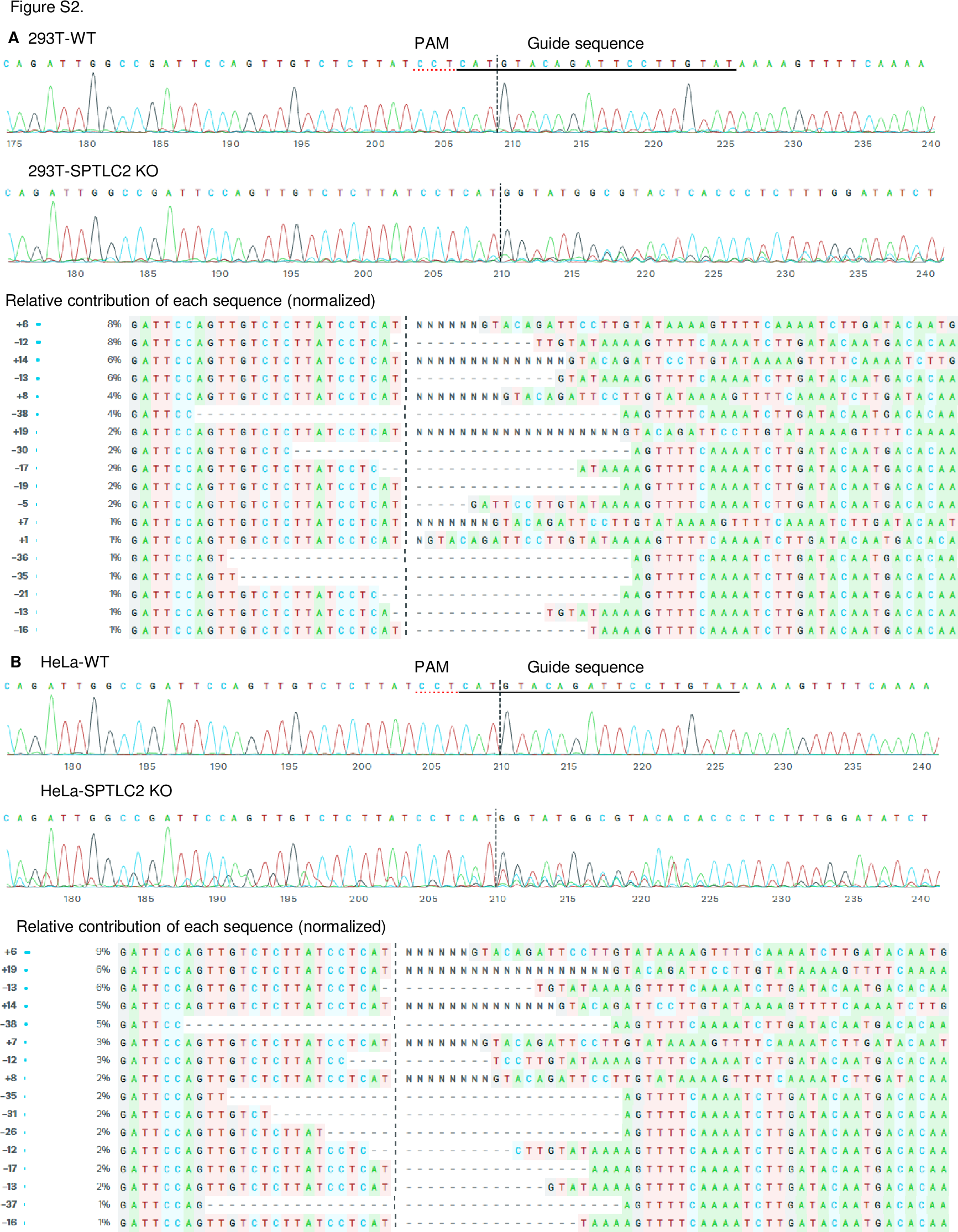

Supplement: S2 Fig — A-B. Sanger sequencing of SPTLC2 in control and knockout 293T or HeLa cells. Sequencing data were analyzed by ICE CRISPR Analysis Tool (https://ice.synthego.com). The guide sequences are represented by a horizontal black underlined region, the PAM sites are shown with a red underline, and the actual cut sites are indicated by a vertical black dotted line. (TIF) [file ppat.1011232.s002.tif]

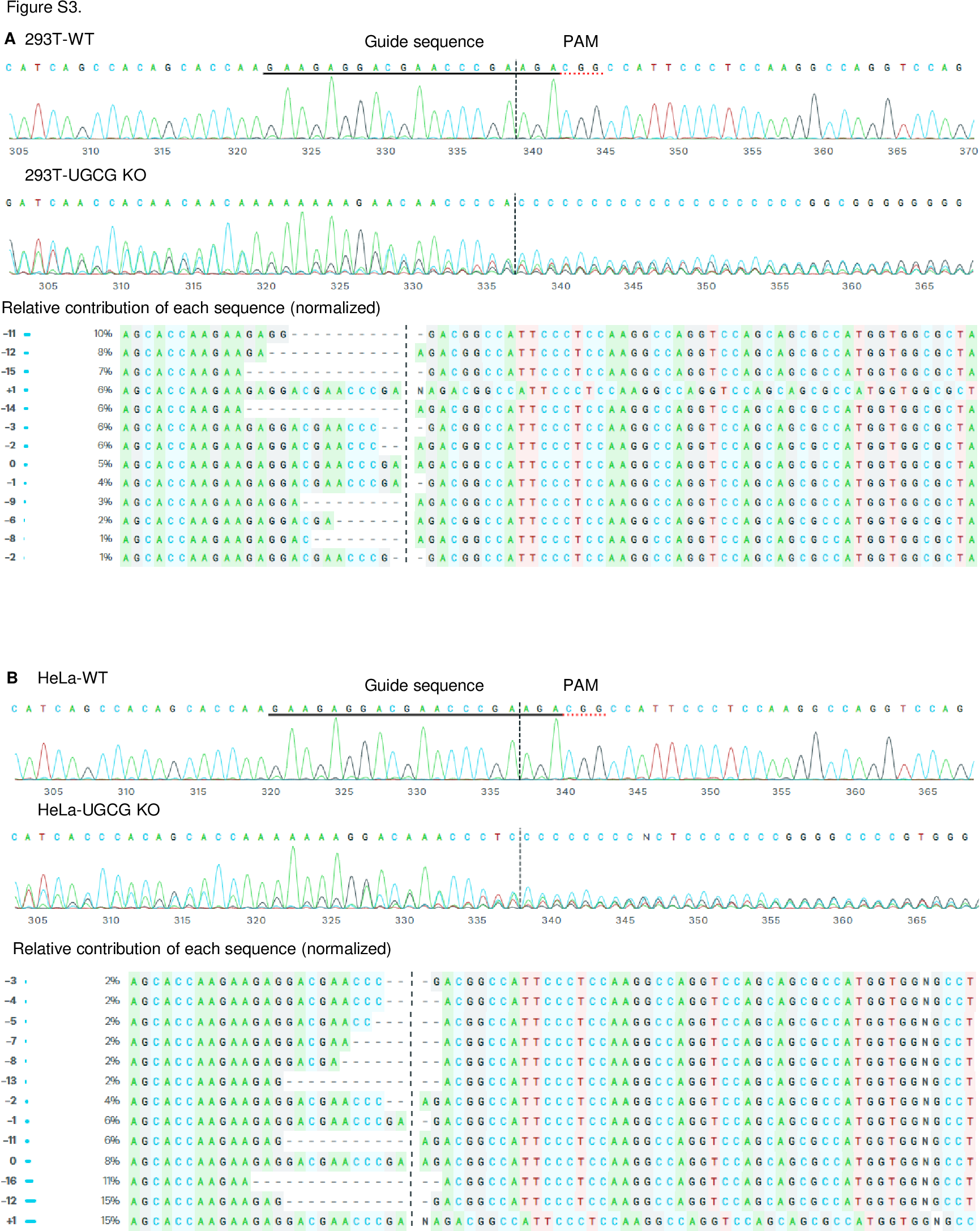

Supplement: S3 Fig — A-B. Sanger sequencing of UGCG in control and knockout 293T or HeLa cells. Sequencing data were analyzed by ICE CRISPR Analysis Tool (https://ice.synthego.com). The guide sequences are represented by a horizontal black underlined region, the PAM sites are shown with a red underline, and the actual cut sites are indicated by a vertical black dotted line. (TIF) [file ppat.1011232.s003.tif]

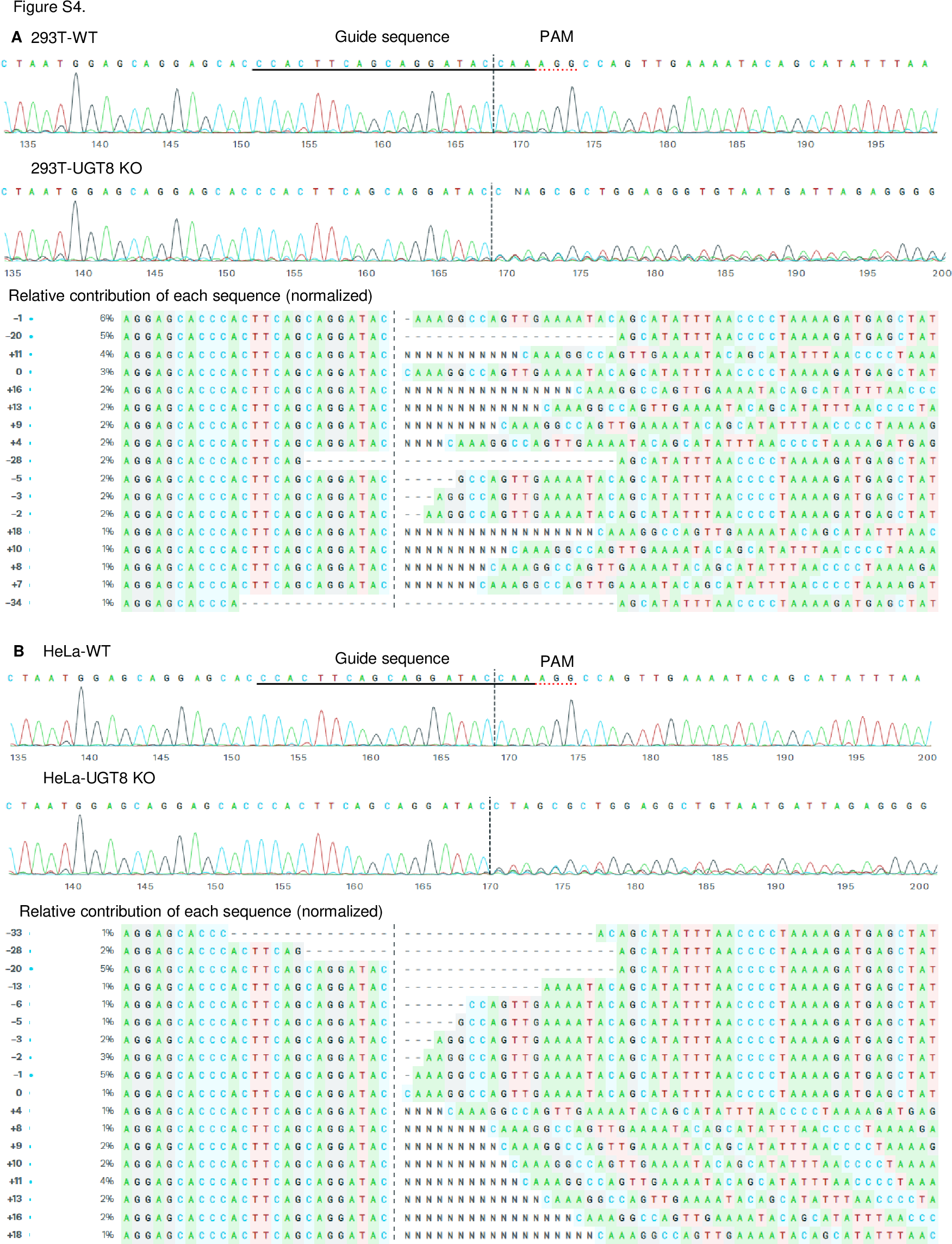

Supplement: S4 Fig — A-B. Sanger sequencing of UGT8 in control and knockout 293T or HeLa cells. Sequencing data were analyzed by ICE CRISPR Analysis Tool (https://ice.synthego.com). The guide sequences are represented by a horizontal black underlined region, the PAM sites are shown with a red underline, and the actual cut sites are indicated by a vertical black dotted line. (TIF) [file ppat.1011232.s004.tif]

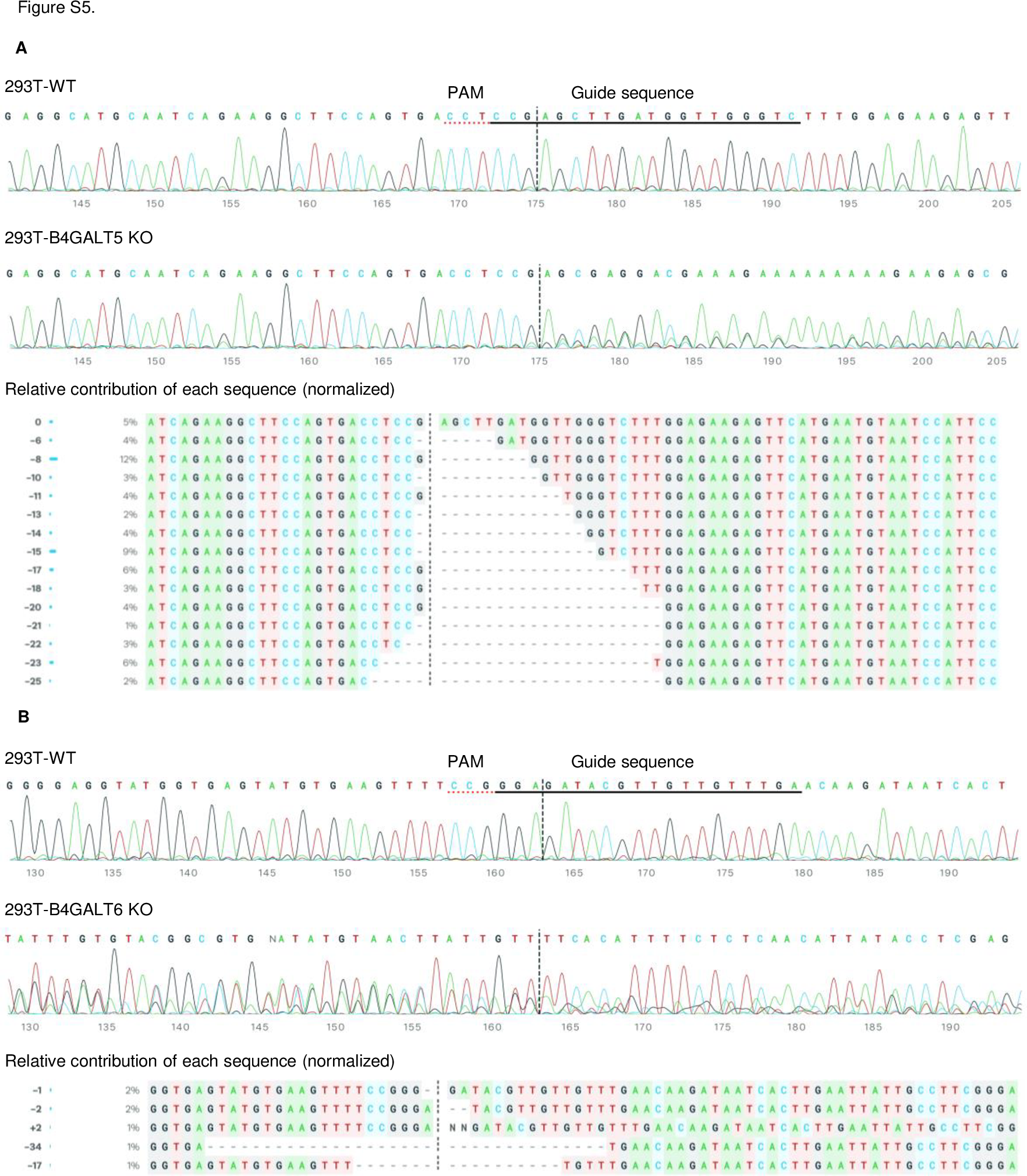

Supplement: S5 Fig — A-B. Sanger sequencing of B4GALT5 or B4GALT6 in control and knockout 293T cells. Sequencing data were analyzed by ICE CRISPR Analysis Tool (https://ice.synthego.com). The guide sequences are represented by a horizontal black underlined region, the PAM sites are shown with a red underline, and the actual cut sites are indicated by a vertical black dotted line. (TIF) [file ppat.1011232.s005.tif]

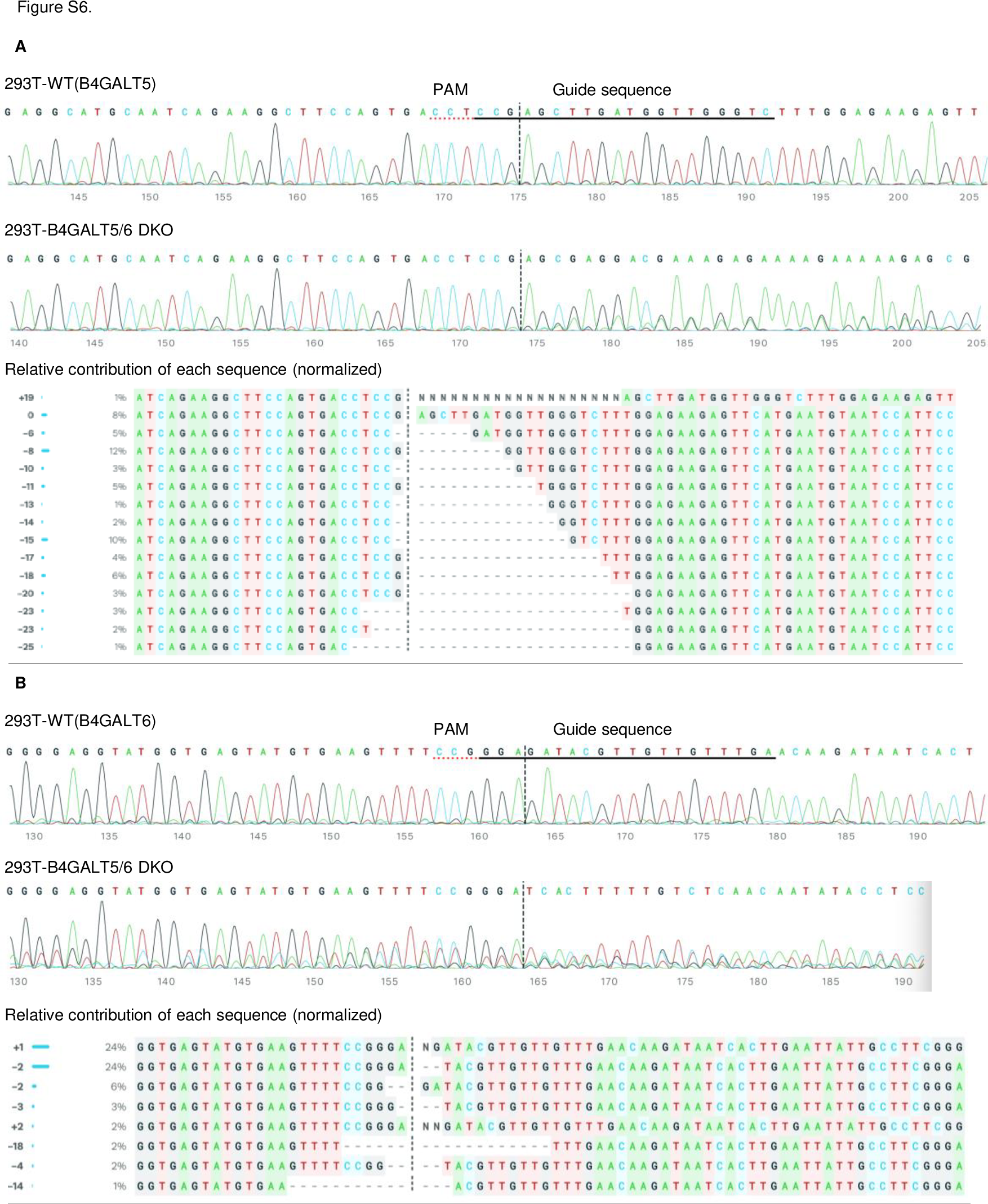

Supplement: S6 Fig — A-B. Sanger sequencing of B4GALT5 or B4GALT6 in control and double knockout 293T cells. Sequencing data were analyzed by ICE CRISPR Analysis Tool (https://ice.synthego.com). The guide sequences are represented by a horizontal black underlined region, the PAM sites are shown with a red underline, and the actual cut sites are indicated by a vertical black dotted line. (TIF) [file ppat.1011232.s006.tif]

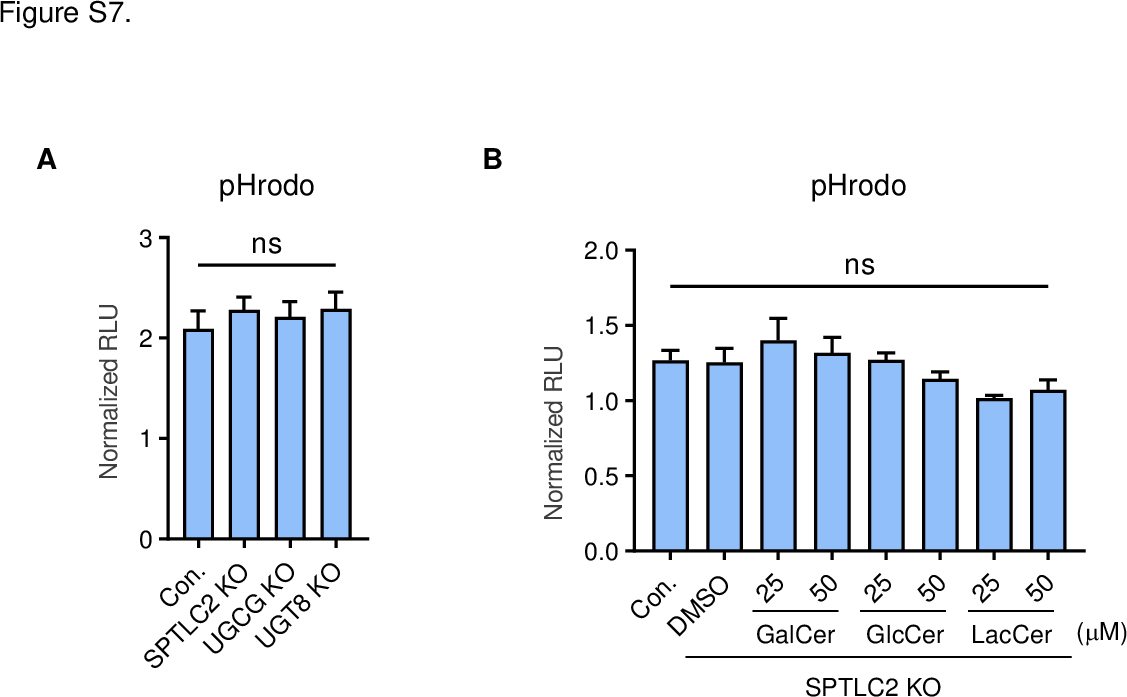

Supplement: S7 Fig — A. Control and KO cells were incubated with pHrodo green dextran for 2 hours and fluorescence was measured with a microplate reader. B. SPTLC2-KO 293T cells were cultured in media supplemented with 50 μM indicated glycosphingolipids for 24 hours and then incubated with pHrodo green dextran for additional 2 hours. Fluorescence was measured with a microplate reader, and NC cells without glycosphingolipids supplement were included for comparison. Data shown are means ± SEM from representative experiments (n = 3 technical replicates). P values were determined by ordinary one-way ANOVA with Dunnett’s multiple comparison tests. (TIF) [file ppat.1011232.s007.tif]

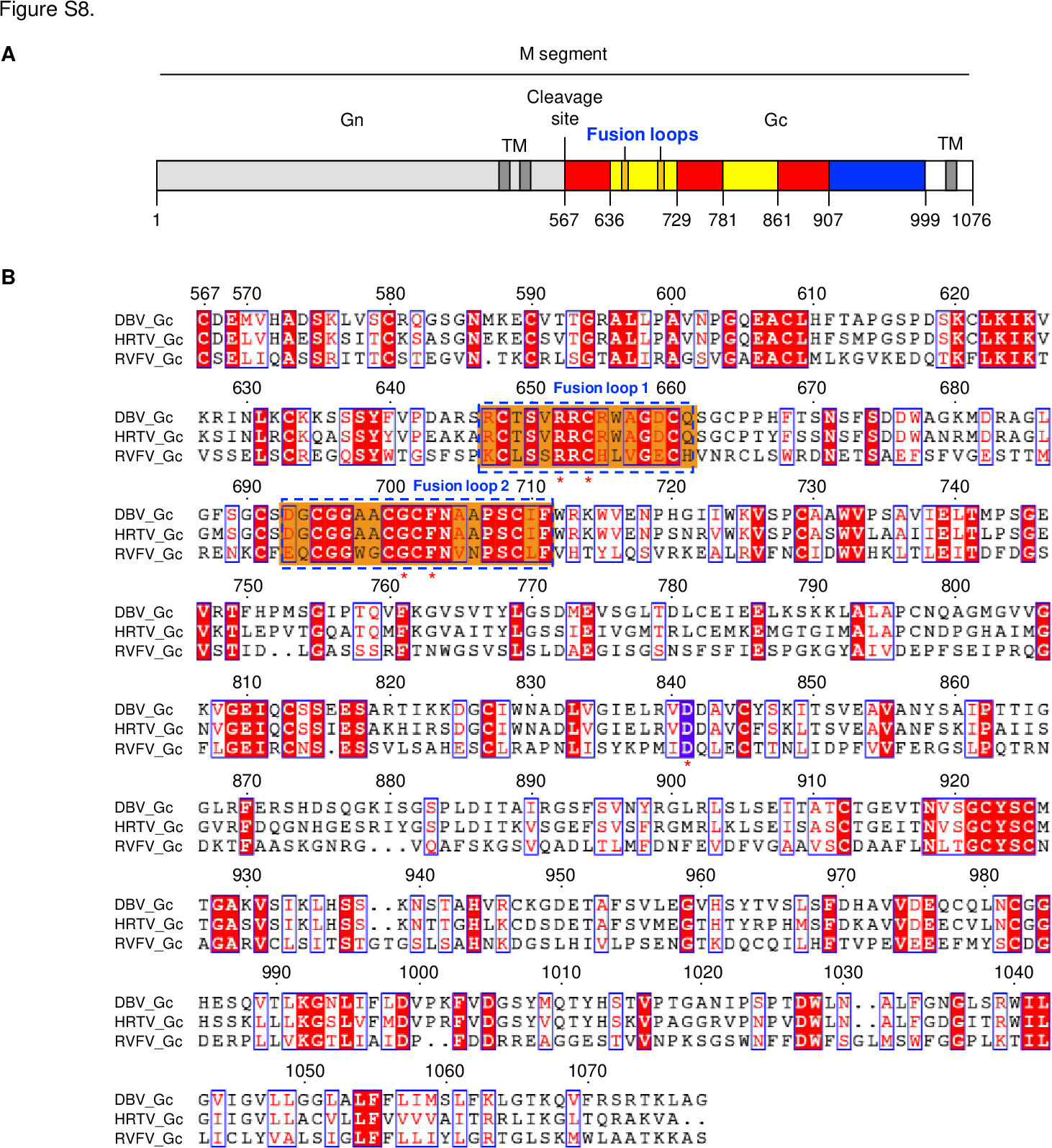

Supplement: S8 Fig — A. Diagram of the HRTV M segment. Gc ectodomain is colored by domains (red, domain I; yellow, domain II; blue, domain III). TM, transmembrane domain. B. Sequence alignment of Gc glycoproteins of HRTV, DBV, and RVFV. The amino acids interacting with GlcCer in HRTV Gc are marked with asterisks. (TIF) [file ppat.1011232.s008.tif]

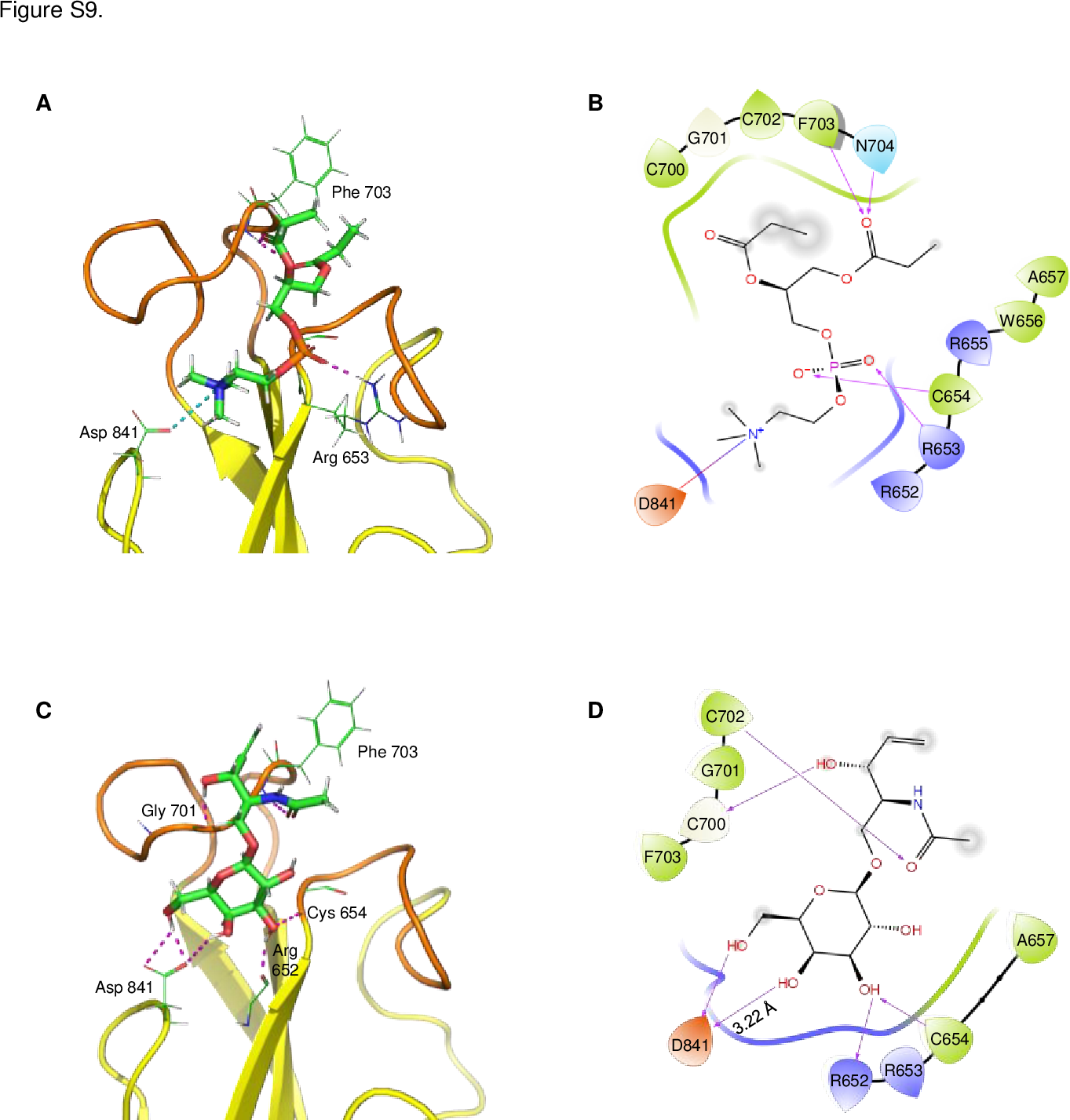

Supplement: S9 Fig — A. Induced-fit docking pose of DOPC with HRTV Gc protein. The hydrogen bonds and salt bridge between DOPC and Gc are colored with magenta and cyan, respectively. B. 2D representation of docking interaction of GlcCer with the binding site residues of HRTV Gc. The hydrogen bonds are shown in magenta lines with arrows and the salt bridge is between residue D841 and the trimethylamine group of DOPC. C. Induced-fit docking pose of GalCer with HRTV Gc protein. D. 2D representation of docking interaction of GalCer with the binding site residues of HRTV Gc. (TIF) [file ppat.1011232.s009.tif]

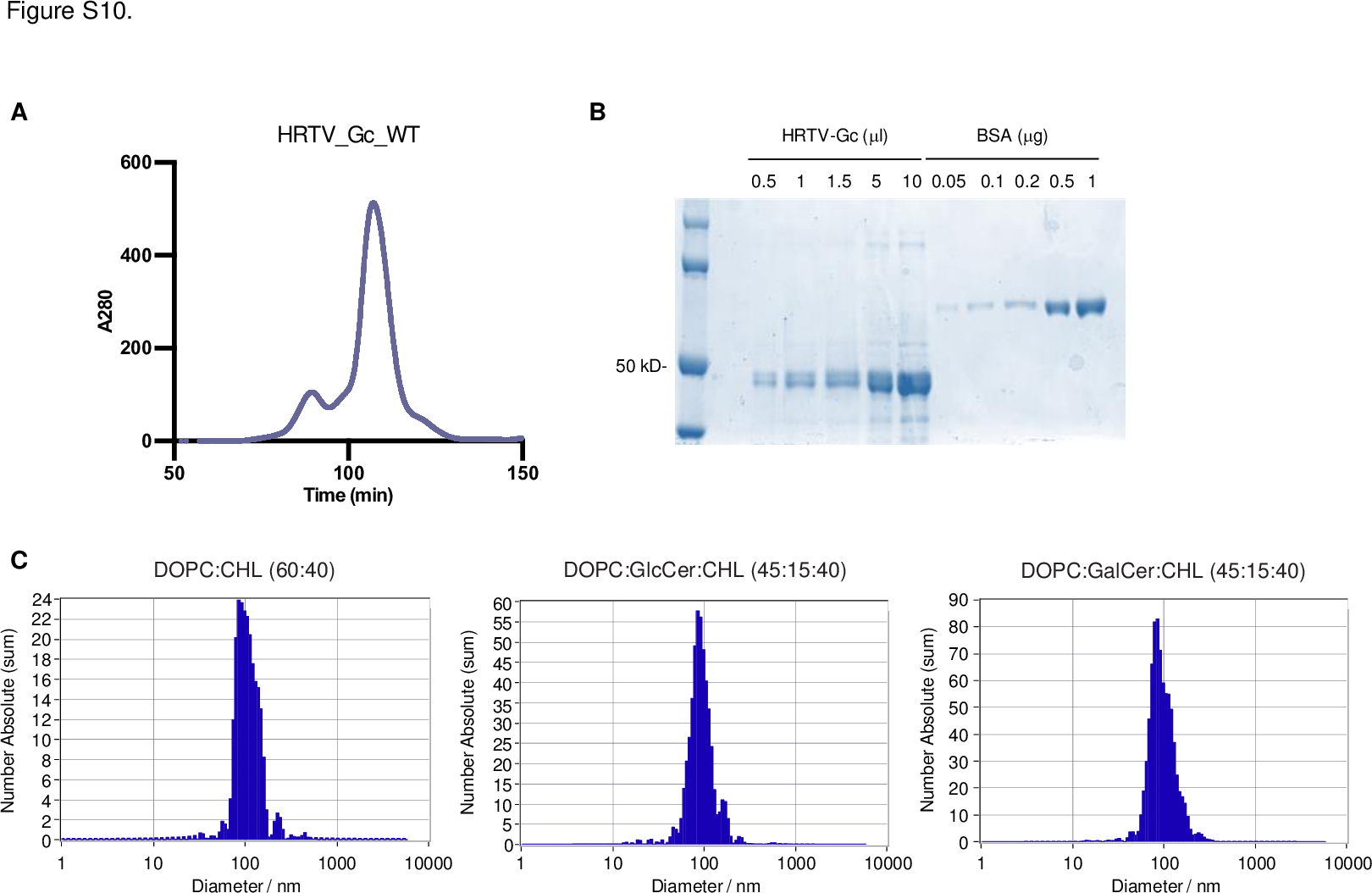

Supplement: S10 Fig — A. Size exclusion chromatography (SEC) analysis of purified HRTV Gc. SEC was performed on a HiLoad 16/600 Superdex 200pg column (Cytiva) equilibrated in 20 mM Tris-HCl (pH 8.0) and 50 mM NaCl. The eluate was analyzed for absorbance at 280 nm. B. Coomassie blue staining of purified HRTV Gc. SDS-PAGE samples collected from SEC were stained by Coomassie blue with the molecular weight (approximately 45 kDa). C. Nanoparticle tracking analysis (NTA) showing the distribution of diameters and size of the indicated liposome. (TIF) [file ppat.1011232.s010.tif]

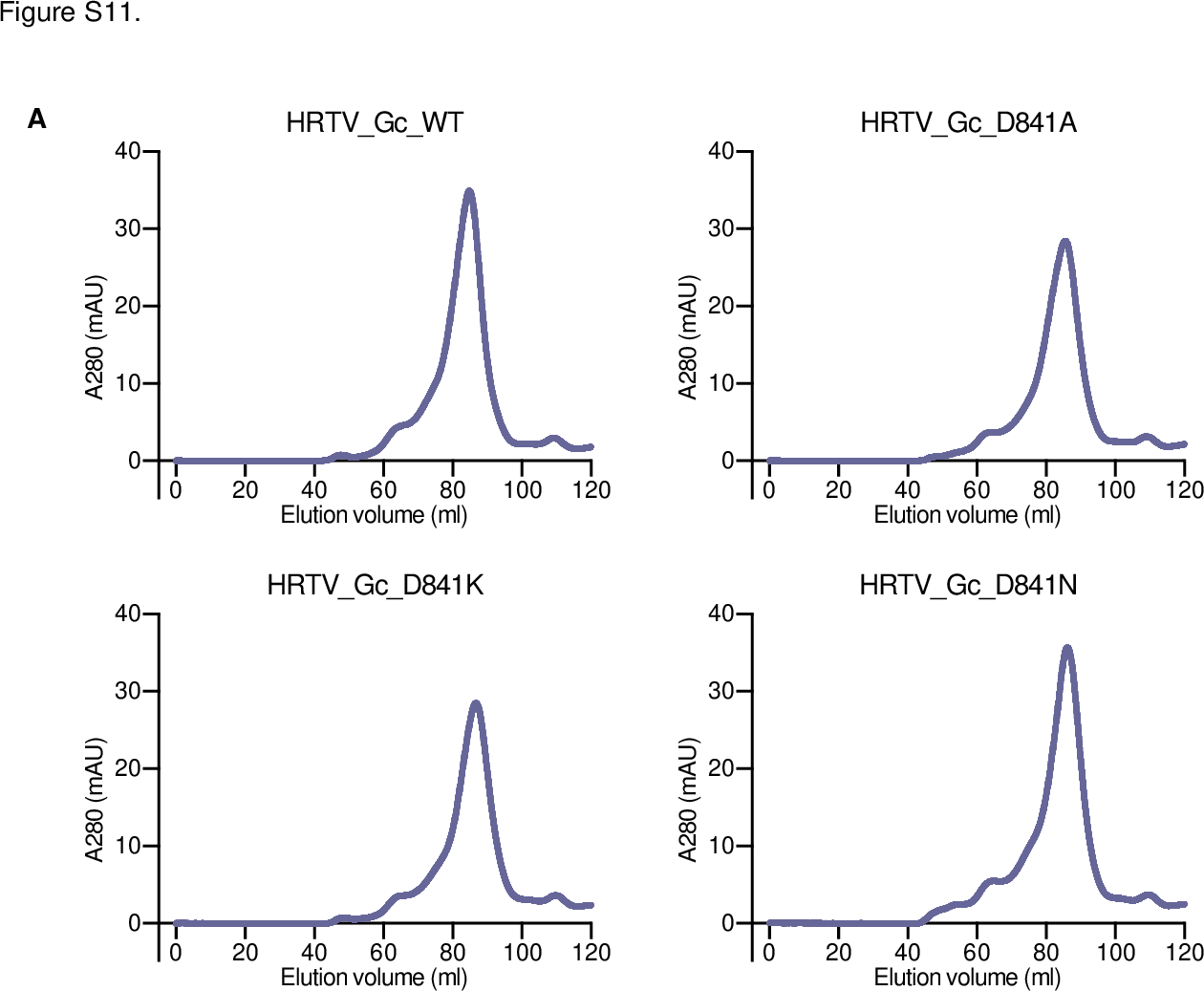

Supplement: S11 Fig — A. SEC analysis of purified HRTV Gc mutant proteins. HRTV Gc WT and mutants were collected from the same fractions for subsequence binding assay. (TIF) [file ppat.1011232.s011.tif]

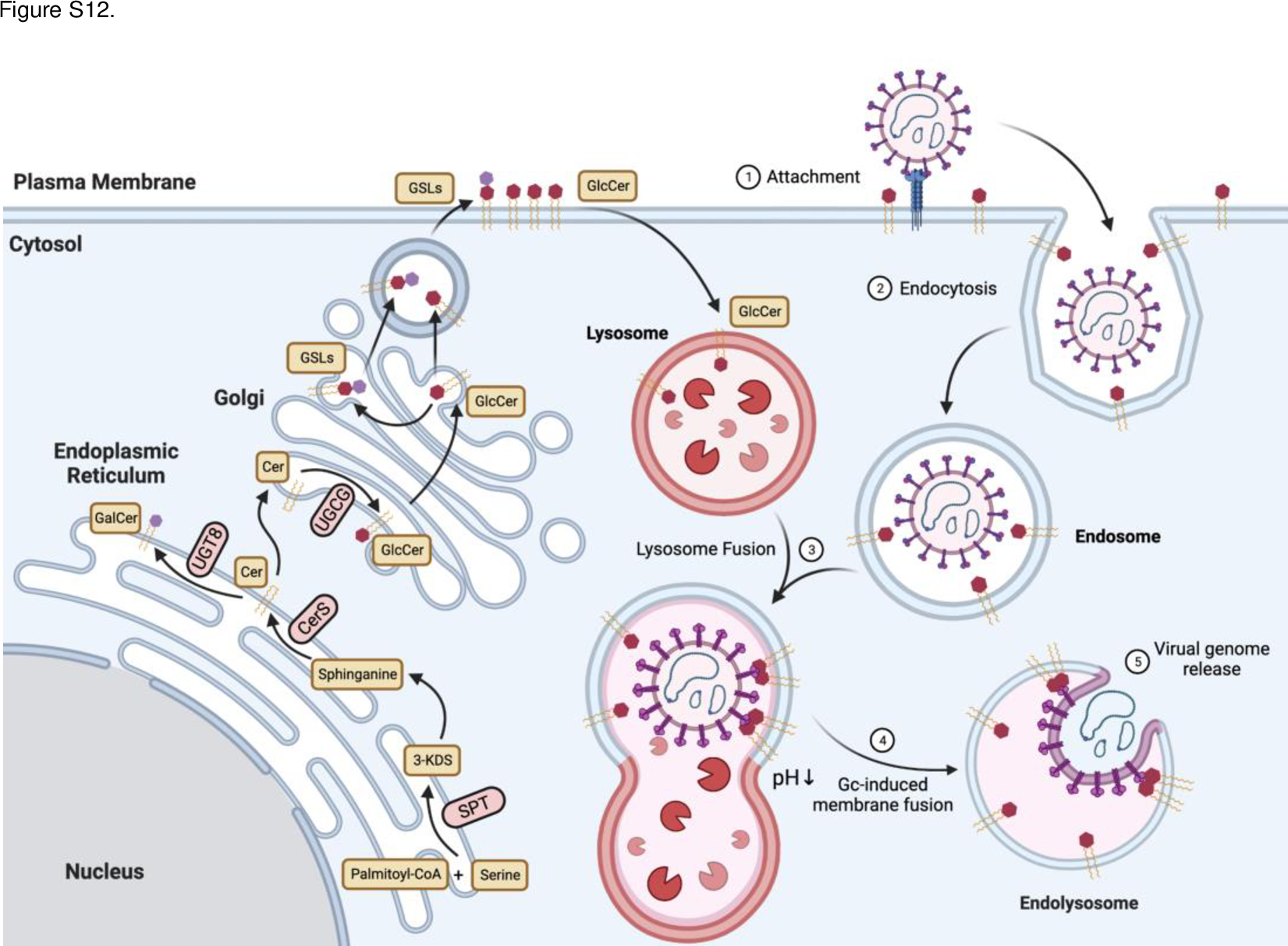

Supplement: S12 Fig — Sphingolipid de novo synthesis pathway is initiated in the ER, where palmitoyl-CoA and serine are catalyzed into ceramide by enzymes, including SPT and CerS. Subsequently, ceramide is converted to GalCer by UGT8 in the ER or directly transported to the Golgi complex. In the Golgi complex, ceramide is further converted into GlcCer by UGCG. GlcCer is then converted to other complex GSLs by adding variable carbohydrate groups. GlcCer and GSLs are delivered to the plasma membrane by vesicular transport and GlcCer in the plasma membrane is subsequently recycled from cell surface and degraded in the lysosome via the endocytic pathway. HRTV infection is triggered by an interaction between viral glycoproteins and host receptor(s) on cell surface. Virus particles are then internalized and enter early endosomes. During viral trafficking, endosome fuses with lysosome, inducing the disassociation of Gn/Gc dimer and the conformational change of Gc protein. Subsequently, Gc protein inserts into target membrane and interacts with GlcCer to form a stable protein-lipid complex, which is essential for an efficient membrane fusion for viral genome releases its genome to the cytosol. 3-KDS, 3-ketodihydrosphingosine; Cer, ceramide; CerS, ceramide synthase; ER, endoplasmic reticulum; GalCer, galactosylceramide; GlcCer, glucosylceramide; GSL, glycosphingolipid; SPT, serine palmitoyltransferase; UGCG, ceramide Glucosyltransferase; UGT8, uridine diphosphate glycosyltransferase 8. Figure created with BioRender.com. (TIF) [file ppat.1011232.s012.tif]
